# Risk factors and disease profile of post-vaccination SARS-CoV-2 infection in UK users of the COVID Symptom Study app: a prospective, community-based, nested, case-control study

**DOI:** 10.1016/S1473-3099(21)00460-6

**Published:** 2022-01

**Authors:** Michela Antonelli, Rose S Penfold, Jordi Merino, Carole H Sudre, Erika Molteni, Sarah Berry, Liane S Canas, Mark S Graham, Kerstin Klaser, Marc Modat, Benjamin Murray, Eric Kerfoot, Liyuan Chen, Jie Deng, Marc F Österdahl, Nathan J Cheetham, David A Drew, Long H Nguyen, Joan Capdevila Pujol, Christina Hu, Somesh Selvachandran, Lorenzo Polidori, Anna May, Jonathan Wolf, Andrew T Chan, Alexander Hammers, Emma L Duncan, Tim D Spector, Sebastien Ourselin, Claire J Steves

**Affiliations:** a School of Biomedical Engineering and Imaging Sciences, King's College London, London, UK; b Department of Twin Research and Genetic Epidemiology, King's College London, London, UK; c Department of Ageing and Health, Guy's and St Thomas' NHS Foundation Trust, London, UK; d Department of Endocrinology, Guy's and St Thomas' NHS Foundation Trust, London, UK; e Diabetes Unit, Massachusetts General Hospital, Boston, MA, USA; f Center for Genomic Medicine, Massachusetts General Hospital, Boston, MA, USA; g Programs in Metabolism, Broad Institute of MIT and Harvard, Cambridge, MA, USA; h Programs in Medical and Population Genetics, Broad Institute of MIT and Harvard, Cambridge, MA, USA; i Department of Medicine, Harvard Medical School, Boston, MA, USA; j MRC Unit for Lifelong Health and Ageing at UCL, University College London, London, UK; k Centre for Medical Image Computing, University College London, London, UK; l Clinical and Translational Epidemiology Unit, Massachusetts General Hospital and Harvard Medical School, Boston, MA, USA; m ZOE, London, UK; n King's College London and Guy's and St Thomas' PET Centre, London, UK

## Abstract

**Background:**

COVID-19 vaccines show excellent efficacy in clinical trials and effectiveness in real-world data, but some people still become infected with SARS-CoV-2 after vaccination. This study aimed to identify risk factors for post-vaccination SARS-CoV-2 infection and describe the characteristics of post-vaccination illness.

**Methods:**

This prospective, community-based, nested, case-control study used self-reported data (eg, on demographics, geographical location, health risk factors, and COVID-19 test results, symptoms, and vaccinations) from UK-based, adult (≥18 years) users of the COVID Symptom Study mobile phone app. For the risk factor analysis, cases had received a first or second dose of a COVID-19 vaccine between Dec 8, 2020, and July 4, 2021; had either a positive COVID-19 test at least 14 days after their first vaccination (but before their second; cases 1) or a positive test at least 7 days after their second vaccination (cases 2); and had no positive test before vaccination. Two control groups were selected (who also had not tested positive for SARS-CoV-2 before vaccination): users reporting a negative test at least 14 days after their first vaccination but before their second (controls 1) and users reporting a negative test at least 7 days after their second vaccination (controls 2). Controls 1 and controls 2 were matched (1:1) with cases 1 and cases 2, respectively, by the date of the post-vaccination test, health-care worker status, and sex. In the disease profile analysis, we sub-selected participants from cases 1 and cases 2 who had used the app for at least 14 consecutive days after testing positive for SARS-CoV-2 (cases 3 and cases 4, respectively). Controls 3 and controls 4 were unvaccinated participants reporting a positive SARS-CoV-2 test who had used the app for at least 14 consecutive days after the test, and were matched (1:1) with cases 3 and 4, respectively, by the date of the positive test, health-care worker status, sex, body-mass index (BMI), and age. We used univariate logistic regression models (adjusted for age, BMI, and sex) to analyse the associations between risk factors and post-vaccination infection, and the associations of individual symptoms, overall disease duration, and disease severity with vaccination status.

**Findings:**

Between Dec 8, 2020, and July 4, 2021, 1 240 009 COVID Symptom Study app users reported a first vaccine dose, of whom 6030 (0·5%) subsequently tested positive for SARS-CoV-2 (cases 1), and 971 504 reported a second dose, of whom 2370 (0·2%) subsequently tested positive for SARS-CoV-2 (cases 2). In the risk factor analysis, frailty was associated with post-vaccination infection in older adults (≥60 years) after their first vaccine dose (odds ratio [OR] 1·93, 95% CI 1·50–2·48; p<0·0001), and individuals living in highly deprived areas had increased odds of post-vaccination infection following their first vaccine dose (OR 1·11, 95% CI 1·01–1·23; p=0·039). Individuals without obesity (BMI <30 kg/m^2^) had lower odds of infection following their first vaccine dose (OR 0·84, 95% CI 0·75–0·94; p=0·0030). For the disease profile analysis, 3825 users from cases 1 were included in cases 3 and 906 users from cases 2 were included in cases 4. Vaccination (compared with no vaccination) was associated with reduced odds of hospitalisation or having more than five symptoms in the first week of illness following the first or second dose, and long-duration (≥28 days) symptoms following the second dose. Almost all symptoms were reported less frequently in infected vaccinated individuals than in infected unvaccinated individuals, and vaccinated participants were more likely to be completely asymptomatic, especially if they were 60 years or older.

**Interpretation:**

To minimise SARS-CoV-2 infection, at-risk populations must be targeted in efforts to boost vaccine effectiveness and infection control measures. Our findings might support caution around relaxing physical distancing and other personal protective measures in the post-vaccination era, particularly around frail older adults and individuals living in more deprived areas, even if these individuals are vaccinated, and might have implications for strategies such as booster vaccinations.

**Funding:**

ZOE, the UK Government Department of Health and Social Care, the Wellcome Trust, the UK Engineering and Physical Sciences Research Council, UK Research and Innovation London Medical Imaging and Artificial Intelligence Centre for Value Based Healthcare, the UK National Institute for Health Research, the UK Medical Research Council, the British Heart Foundation, and the Alzheimer's Society.

## Introduction

Vaccination against SARS-CoV-2 is a leading strategy to change the course of the COVID-19 pandemic worldwide. The UK was the first country to authorise a vaccine against SARS-CoV-2, with three licensed as of July, 2021: BNT162b2 (tozinameran; Pfizer–BioNTech), mRNA-1273 (elasomeran; Moderna), and ChAdOx1 nCoV-19 (Oxford–AstraZeneca), each with good efficacy in phase 3 clinical trials.^1^, ^2^, ^3^, ^4^ Of the 67·1 million adults in the UK, by July 4, 2021, around 45·4 million people had received one vaccine dose and around 33·7 million people had received two doses.^5^ UK data present an early insight into the real-world effectiveness of COVID-19 vaccines and the remaining challenges post-vaccination.

A previous analysis of community-based individuals in the COVID Symptom Study showed a significant reduction in infection post-vaccination from 12 days after the first dose,^6^ findings that were recapitulated in a UK-based, real-world, case-control study.^7^ National surveillance data from the first 4 months of Israel's vaccination campaign showed that two doses of BNT162b2 reduced both symptomatic and asymptomatic infections, COVID-19-related hospitalisations, severe disease, and death.^8^

Nonetheless, some people still contract COVID-19 after vaccination, and further virus variants could evolve with increased transmissibility (as with B.1.1.7 [the alpha variant]).^9^ Indeed, variants of concern have shown reduced neutralisation by convalescent and post-vaccination serum samples in vitro,^10^ and led to increased rates of post-vaccination infection compared with the original outbreak variant in early findings from a real-world case-control study.^11^ Data suggest that although COVID-19 is usually milder if contracted after vaccination than in unvaccinated individuals, mortality remains high in hospitalised individuals: data from the International Severe Acute Respiratory and Emerging Infection Consortium have shown a mortality of 27·0% (400 of 1482 died) in individuals hospitalised with COVID-19 in the UK more than 21 days after vaccination, similar to mortality rates observed during the first wave (March–April, 2020).^12^, ^13^

**Research in context**

**Evidence before this study**

To identify existing evidence of risk factors for, and the characteristics of, post-vaccination SARS-CoV-2 infection, we searched PubMed for peer-reviewed articles published between Dec 1, 2020, and July 4, 2021, using the keywords (“COVID-19” OR “SARS-CoV-2”) AND (“vaccine” OR “vaccination”) AND (“infection”) AND (“risk factor*” OR “characteristic*” OR “symptom*”). We did not restrict our search by language or type of publication. Of the 662 published PubMed articles identified, we found one paper assessing risk factors for post-vaccination infection in fully vaccinated US veterans. This paper suggested that age and the presence of anaemia were positively associated, and being Black was negatively associated, with post-vaccination infection. We found no studies looking at risk factors following a single dose of vaccine or the disease profile in vaccinated, community-based individuals. Previous studies in unvaccinated populations have shown that social and occupational factors influence the risk of SARS-CoV-2 infection, and that personal factors (eg, age, male sex, multiple morbidities, and frailty) increase the risk for adverse outcomes in COVID-19. Phase 3 clinical trials have shown good efficacy for BNT162b2 (tozinameran; Pfizer–BioNTech), ChAdOx1 nCoV-19 (Oxford–AstraZeneca), and mRNA-1273 (elasomeran; Moderna) vaccines against SARS-CoV-2 infection, supported by published real-world data, with vaccines also reducing the risk of adverse outcomes, including hospitalisation and death. We found two published studies of in-vitro and early real-world evidence suggesting that BNT162b2 and ChAdOx1 nCoV-19 vaccines might be less effective against emerging variants of concern than against the original outbreak variant.

**Added value of this study**

To our knowledge, this observational study was the first to investigate the characteristics of SARS-CoV-2 infection after first and second COVID-19 vaccinations. Vaccination (compared with no vaccination) was associated with reduced odds of hospitalisation or having more than five symptoms in the first week of illness following the first or second dose, and long-duration (≥28 days) symptoms following the second dose. Almost all symptoms were reported less frequently in infected vaccinated than in infected unvaccinated individuals, and vaccinated participants were more likely to be completely asymptomatic, especially if they were 60 years or older. In our risk factor analysis, we found that frailty was associated with post-vaccination infection in older adults (≥60 years) after their first vaccine dose. Adverse determinants of health, such as living in highly deprived areas and obesity, were associated with an increased likelihood of SARS-CoV-2 infection following the first vaccine dose.

**Implications of all the available evidence**

Some individuals still become infected with SARS-CoV-2 after vaccination; our data suggest that frail, older adults and those living in more deprived areas are at increased risk. However, COVID-19 appears to be less severe in vaccinated versus unvaccinated individuals. Our results are relevant for health policies post-vaccination and highlight the need to balance personal protective measures in those at risk of post-vaccination infection with the adverse effects from ongoing social restrictions. Strategies, such as timely prioritisation of booster vaccinations and optimised infection control measures, could be considered for at-risk groups. Research is also needed on how to enhance the immune response to vaccination in those at higher risk of post-vaccination infection.

Identifying and protecting individuals at increased risk of post-vaccination infection is becoming increasingly salient as more people are vaccinated. Groups at increased risk of SARS-CoV-2 infection before vaccines became available included frontline health-care workers and individuals from areas of greater relative deprivation (probably reflecting increased exposure),^14^, ^15^ and increasing age, male sex, multimorbidity, and frailty are associated with poorer COVID-19 outcomes.^16^, ^17^, ^18^ A study of fully vaccinated US veterans showed that older age and the presence of anaemia were positively associated with post-vaccination infection, and Black individuals were at a lower risk than White individuals.^19^ However, this study was done in a fully vaccinated, older (median age 73 years [IQR 68–78]), and predominantly male cohort, and did not assess lifestyle and sociodemographic risk factors for post-vaccination infection.

Individuals with COVID-19 have differing symptoms and clinical needs.^20^ Elucidating symptom profiles in individuals with COVID-19 after vaccination has clinical utility, facilitating the identification of risk groups for intervention, predicting medical resource requirements, and informing appropriate testing guidelines. Additionally, some unvaccinated individuals with COVID-19 have prolonged illness duration (so-called long COVID),^21^ and whether vaccination reduces the risk of long COVID is currently unknown.

Therefore, we aimed to (1) describe individual risk factors associated with SARS-CoV-2 infection at least 14 days after first vaccination or 7 days after second vaccination, and (2) assess illness duration, severity, and symptom profile in individuals with SARS-CoV-2 infection after their first and second vaccinations, compared with unvaccinated individuals with SARS-CoV-2 infection.

## Methods

### Study design and participants

This prospective, community-based, nested, case-control study used data from UK-based, adult (≥18 years) participants of the COVID Symptom Study logged through a free mobile phone app developed by ZOE (London, UK) and King's College London (London, UK).^22^ The app was launched in the UK on March 24, 2020, and by July 4, 2021, had nearly 4·5 million unique participants providing data by self-report or proxy report. At registration, each participant reported baseline demographic information (eg, age, sex, ethnicity, weight, height, and health-care worker status), geographical location, and information on health risk factors, including comorbidities, lifestyle, frailty, visits to hospital, and adherence to mask-wearing guidance. Participants were encouraged to self-report any of 32 prespecified symptoms (appendix p 1) daily, providing prospective longitudinal information on incident symptoms. Those with new symptoms were prompted to book and take a SARS-CoV-2 test. All users were encouraged to record any SARS-CoV-2 testing results (whether prompted by the app or otherwise), and, from Dec 11, 2020, any SARS-CoV-2 vaccination and subsequent symptoms.^22^ Users with missing or inconsistent information were excluded from our analysis. The inclusion process for cases and controls is shown in the appendix (p 14).

Cases had received a first or second dose of a COVID-19 vaccine since Dec 8, 2020; had either a positive RT-PCR test or lateral flow antigen test (LFAT) at least 14 days after their first vaccination (but before their second; cases 1) or a positive RT-PCR test or LFAT at least 7 days after their second vaccination (cases 2); and had no positive SARS-CoV-2 test before vaccination. If more than one positive test result was reported, only the first positive test was selected. To identify risk factors for post-vaccination infection, we selected two control groups among the vaccinated (since Dec 8, 2020) UK-based adult users of the COVID Symptom Study app who had not tested positive for SARS-CoV-2 before vaccination: a control group of users reporting a negative RT-PCR test or LFAT at least 14 days after their first vaccination but before their second (controls 1) and a control group of users reporting a negative RT-PCR test or LFAT at least 7 days after their second vaccination (controls 2). Controls 1 and controls 2 were matched (1:1) with cases 1 and cases 2, respectively, by use of the date of the post-vaccination COVID-19 test, health-care worker status, and sex. If multiple negative tests were reported, the last test date was used for matching.

To compare the disease profile of SARS-CoV-2 infection before and after vaccination, we sub-selected participants from cases 1 and cases 2 who had used the app for at least 14 consecutive days after testing positive for SARS-CoV-2 (denoted as cases 3 and cases 4, respectively), so that symptoms of infection could be assessed. Controls for the disease profile analysis were those who reported a SARS-CoV-2-positive RT-PCR test or LFAT, were unvaccinated until data censoring, and had used the app for at least 14 consecutive days after the test. Among these users, two groups were formed: controls 3 and controls 4, matching (1:1) with cases 3 and cases 4, respectively, by the date of the positive COVID-19 test, health-care worker status, sex, body-mass index (BMI), and age. Individuals in all case and control groups who did not report an RT-PCR or LFAT test after Dec 8, 2020, were excluded.

For all control groups, we used a matching algorithm based on minimum Euclidean distance^23^ between the vectors of the covariates, with age, BMI, and the date of the test as numerical variables, and sex as a binary variable multiplied by 100 to ensure balance between covariate strengths. We considered health-care worker status, coded as a a categorical variable in the app, as a numerical variable (0=not a health-care worker; 1=health-care worker who does not interact with patients; 2=health-care worker who does not treat patients; 3=health-care worker who interacts with patients; 4=health-care worker who treats patients). Participants could only choose one of these options.

All app users provided digital informed consent for data usage for COVID-19-related research. In the UK, the app and the study were approved by King's College London's ethics committee (REMAS number 18210; reference LRS-19/20–18210).

### Risk factor variable definitions

For this analysis, the outcome variable was case status (a self-reported positive RT-PCR test or LFAT for SARS-CoV-2). We considered a priori-defined risk factors for SARS-CoV-2 infection based on previous evidence in unvaccinated individuals:^16^, ^17^, ^18^ age; BMI; self-reported comorbidities (ie, cancer, diabetes, asthma, lung disease, heart disease, and kidney disease), analysed individually as binary variables; dependency level (frailty) assessed by the PRISMA-7 questionnaire, which is embedded in app registration,^24^, ^25^ as a binary variable (PRISMA-7 score ≥3 defined as frail and <3 defined as not frail);^26^ local area Index of Multiple Deprivation (IMD; a score ranging from 1 [most deprived] to 10 [least deprived] estimating relative locality deprivation derived from postal code and lower layer super output area) divided into low IMD (1–3), intermediate IMD (4–7), and high IMD (8–10) groups;^27^ and four healthy lifestyle factors (no current smoking, no obesity [BMI <30 kg/m^2^], physical activity at least once per week [non-sedentary], and a healthy diet pattern; appendix p 17). We also calculated a healthy lifestyle score on the basis of these four lifestyle factors,^28^ by which participants received 1 point for each healthy lifestyle factor and the sum of the scores gave a total healthy lifestyle score ranging from 0 to 4, with higher scores indicating a healthier lifestyle (appendix p 17).

### Disease severity, duration, and symptom definitions

To compare the disease profile in vaccinated versus unvaccinated individuals testing positive for SARS-CoV-2, we assessed disease severity (asymptomatic or symptomatic; more than five symptoms or five or fewer symptoms reported in the first week of illness;^20^ and self-reported presentation to hospital or no hospital presentation), illness duration (duration of <28 days or ≥28 days), and individual symptom reports. Vaccination status was the exposure. For cases, controls 3, and controls 4, symptoms were considered within a window between 3 days before the COVID-19 test date and up to 14 days after the test date (appendix p 1). This window was used because it might have taken up to 3 days to request an RT-PCR test and receive a result following symptom onset, and symptoms can occur up to 14 days following SARS-CoV-2 exposure.^29^

### Statistical analysis

Data were extracted and pre-processed by use of ExeTera13, a Python library developed at King's College London (version 0.5.5)^30^ that is openly available on GitHub. Statistical analyses used Python 3.7 and the packages NumPy (version 1.19.2), Pandas (version 1.1.3), SciPy (version 1.5.2), and statsmodels (version 0.12.1).

In the risk factor analysis, we assessed the differences in proportions and means of covariates between cases and respective controls using Fisher's exact test for categorical variables and Wilcoxon's test for continuous variables. p values of 0·05 or less were considered statistically significant. Univariate logistic regression models (adjusted for age, BMI, and sex) and multivariate logistic regression models of age and BMI (adjusted for sex) were used to analyse the associations between risk factor variables and post-vaccination infection. Because associated factors might differ by age group, analyses were stratified by sex (male and female) and age (younger adults were those aged 18–59 years and older adults were those aged ≥60 years). To examine whether health-conscious behaviours might explain the association between lifestyle factors and post-vaccination infection, we further adjusted models for reported individual adherence to mask-wearing guidance from June 12, 2020, to Sept 29, 2020. Multivariate logistic regression (adjusted for age, BMI, and sex) was used to assess the independence of frailty, IMD category, and the four healthy lifestyle factors.

For sensitivity analyses, we examined models via inverse probability weighting^31^ to check for potential index event bias of vaccination using weights derived from probabilities of being vaccinated in the population tested and active on the app during the study period (appendix p 15). Data from 1 531 762 app users reporting an RT-PCR or LFAT test within the study period were processed to obtain weights for inverse probability weighting of being vaccinated. Weights were estimated from a logistic regression model for predicting vaccination, which included confounders known to be associated with vaccination status: frailty, IMD, and the comorbidities of cancer, diabetes, lung disease, heart disease, kidney disease, and asthma.

In the disease profile analysis, univariate logistic regression models adjusted by age, BMI, and sex were used to assess the association of individual symptoms, overall illness duration, and disease severity (outcomes) with vaccination status (exposure). Symptoms were examined if they were reported by more than 1% of app users reporting a positive test. We also provide models that were adjusted for frailty and the presence of at least one comorbidity, given the association of these factors with the exposure (vaccination) and outcome (symptoms), which could confound observed associations.

For all regression analyses, odds ratios (ORs) and 95% CIs were calculated. Analyses were not corrected for multiple testing. This study reports on vaccination with BNT162b2, ChAdOx1 nCoV-19, and mRNA-1273 only, as there were no positive cases among the few people who had received other vaccines.

### Role of the funding source

The funders of the study had no role in study design, data analysis, data interpretation, or writing of the report. ZOE, funded by the UK Department of Health and Social Care, made the COVID Symptom Study app available for data collection as a not-for-profit endeavour. Representatives of ZOE approved the final manuscript for submission.

## Results

Between Dec 8, 2020, and July 4, 2021 (date of data census), 1 240 009 app users reported a first dose (442 752 with BNT162b2, 750 137 with ChAdOx1 nCoV-19, and 17 958 with mRNA-1273) and 971 504 reported a second dose (330 760 with BNT162b2, 625 088 with ChAdOx1 nCoV-19, and 3417 with mRNA-1273) of a COVID-19 vaccine. 6030 (0·5%) of 1 240 009 app users reported testing positive for SARS-CoV-2 at least 14 days after their first vaccination but before their second (cases 1) and 2370 (0·2%) of 971 504 reported testing positive at least 7 days after their second dose (cases 2). Users were infected with SARS-CoV-2 a mean of 73 days (SD 44; median 67 days [IQR 33–106]) after their first vaccination and a mean of 51 days (SD 30; median 44 days [29–68]) after their second vaccination (appendix p 16).

In the risk factor analysis, positive and negative tests were confirmed by RT-PCR in around 70% of both cases and controls after their first vaccine dose (appendix p 1). After the second vaccine dose, controls reported a higher proportion of tests by RT-PCR (2020 [85·2%] of 2370) than did cases (1570 [66·2%] of 2370; appendix p 1). There was a higher proportion of female participants than male participants in all groups in the risk factor analysis, cases were significantly younger (p<0·0001) than their respective control groups, and participants in cases 1 had a significantly higher BMI (p=0·0074) than did participants in controls 1 (table 1).

**Table 1:** Characteristics of case participants with COVID-19 after their first or second vaccination and vaccinated control participants without COVID-19 in the risk factor analysis

		**Cases 1**			**Cases 2**			**Controls 1**			**Controls 2**		
		Total (n=6030)	18–59 years (n=3931)	≥60 years (n=2099)	Total (n=2370)	18–59 years (n=1336)	≥60 years (n=1034)	Total (n=6030)	18–59 years (n=4196)	≥60 years (n=1834)	Total (n=2370)	18–59 years (n=1499)	≥60 years (n=871)
**Demographics**													
Sex													
	Female	3766 (62·5%)	2565 (65·3%)	1201 (57·2%)	1451 (61·2%)	882 (66·0%)	569 (55·0%)	3766 (62·5%); p=1·0	2764 (65·9%) p=0·66	1002 (54·6%) p=1·0	1451 (61·2%) p=1·0	971 (64·8%) p=0·49	480 (55·1%) p=0·96
	Male	2264 (37·5%)	1366 (34·7%)	898 (42·8%)	919 (38·8%)	454 (34·0%)	465 (45·0%)	2264 (37·5%)	1432 (34·1%)	832 (45·4%)	919 (38·8%)	528 (35·2%)	391 (44·9%)
Age, years		50·2 (14·1)	42·3 (9·5)	65·0 (8·4)	52·9 (13·5)	43·9 (9·9)	64·5 (7·4)	51·7 (14·5); p<0·0001	44·2 (9·6); p<0·0001	68·9 (7·1); p<0·0001	54·0 (13·1); p<0·0001	46·2 (9·3); p<0·0001	67·4 (5·8); p<0·0001
Body-mass index, kg/m^2^		27·0 (6·5)	27·0 (6·6)	27·0 (6·4)	26·9 (6·7)	27·2 (7·0)	26·6 (6·2)	26·7 (6·9); p=0·0074	26·9 (7·1); p=0·20	26·2 (6·4); p=0·37	26·7 (6·8); p=0·77	27·0 (7·3); p=0·34	26·2 (6·0); p=0·16
Health-care worker		792 (13·1%)	563 (14·3%)	229 (10·9%)	226 (9·5%)	171 (12·8%)	55 (5·3%)	791 (13·1%); p=1·0	688 (16·4%); p=0·0098	103 (5·6%); p<0·0001	226 (9·5%); p=1·0	189 (12·6%); p=0·82	37 (4·2%); p=0·20
**Vaccine type**													
BNT162b2		2478 (41·1%)	1610 (41·0%)	868 (41·4%)	779 (32·9%)	415 (31·1%)	364 (35·2%)	2707 (44·9%); p<0·0001	1848 (44·0%); p=0·0050	859 (46·8%); p=0·0006	893 (37·7%); p<0·0001	538 (35·9%); p=0·0016	355 (40·8%); p=0·0004
ChAdOx1 nCoV-19		3359 (55·7%)	2155 (54·8%)	1204 (57·4%)	1557 (65·7%)	896 (67·1%)	661 (63·9%)	3046 (50·5%); p<0·0001	2104 (50·1%); p<0·0001	942 (51·4%); p=0·0002	1411 (59·5%); p<0·0001	903 (60·2%); p=0·0026	508 (58·3%); p=0·0004
mRNA-1273		86 (1·4%)	86 (2·2%)	0	1 (<0·1%)	1 (0·1%)	0	101 (1·7%); p=0·30	100 (2·4%); p=0·60	1 (0·1%);p=0·46	41 (1·7%); p=0·62	41 (2·7%); p=0·62	0*
Not sure		107 (1·8%)	80 (2·0%)	27 (1·3%)	33 (1·4%)	24 (1·8%)	9 (0·9%)	176 (2·9%); p<0·0001	144 (3·4%); p=0·0001	32 (1·7%); p=0·24	25 (1·1%); p=0·70	17 (1·1%); p=0·54	8 (0·9%); p=1·0
**Comorbidities**													
Cancer		65 (1·1%)	14 (0·4%)	51 (2·4%)	23 (1·0%)	8 (0·6%)	15 (1·5%)	85 (1·4%); p=0·12	16 (0·4%); p=1·0	69 (3·8%); p=0·016	29 (1·2%); p=0·15	5 (0·3%);p=1·0	24 (2·8%); p=0·029
Diabetes		172 (2·9%)	57 (1·5%)	115 (5·5%)	78 (3·3%)	30 (2·2%)	48 (4·6%)	204 (3·4%); p=0·10	96 (2·3%); p=0·0055	108 (5·9%); p=0·58	76 (3·2%); p=0·21	30 (2·0%); p=0·38	46 (5·3%); p=0·26
Lung disease		645 (10·7%)	412 (10·5%)	233 (11·1%)	282 (11·9%)	169 (12·6%)	113 (10·9%)	627 (10·4%); p=0·61	470 (11·2%); p=0·30	157 (8·6%); p=0·0087	249 (10·5%); p=0·82	169 (11·3%); p=0·82	80 (9·2%); p=0·37
Heart disease		208 (3·4%)	34 (0·9%)	174 (8·3%)	87 (3·7%)	16 (1·2%)	71 (6·9%)	228 (3·8%); p=0·35	48 (1·1%); p=0·22	180 (9·8%); p=0·10	92 (3·9%); p=0·33	21 (1·4%); p=0·099	71 (8·2%); p=0·49
Kidney disease		61 (1·0%)	23 (0·6%)	38 (1·8%)	27 (1·1%)	12 (0·9%)	15 (1·5%)	54 (0·9%); p=0·57	27 (0·6%); p=0·78	27 (1·5%); p=0·45	17 (0·7%); p=0·57	8 (0·5%);p=1·0	9 (1·0%); p=0·41
Asthma		883 (14·6%)	600 (15·3%)	283 (13·5%)	385 (16·2%)	242 (18·1%)	143 (13·8%)	851 (14·1%); p=0·42	649 (15·5%); p=0·80	202 (11·0%); p=0·020	318 (13·4%); p=0·045	219 (14·6%); p=0·52	99 (11·4%); p=0·11
Frailty		260 (4·3%)	70 (1·8%)	190 (9·1%)	90 (3·8%)	31 (2·3%)	59 (5·7%)	301 (5·0%); p=0·080	105 (2·5%); p=0·027	196 (10·7%); p=0·086	95 (4·0%); p=0·034	32 (2·1%); p=0·20	63 (7·2%); p=0·031
Presence of at least one comorbidity		1322 (21·9%)	731 (18·6%)	591 (28·2%)	582 (24·6%)	307 (23·0%)	275 (26·6%)	1349 (22·4%); p=0·57	817 (19·5%); p=0·32	532 (29·0%); p=0·57	504 (21·3%); p=0·50	275 (18·3%); p=0·62	229 (26·3%); p=0·80
**Index of Multiple Deprivation**													
Low (1–3)		1216 (20·2%)	819 (20·8%)	397 (18·9%)	436 (18·4%)	261 (19·5%)	175 (16·9%)	1053 (17·5%); p=0·0002	793 (18·9%); p=0·030	260 (14·2%); p=0·013	393 (16·6%); p=0·53	287 (19·1%); p=0·63	106 (12·2%); p=0·023
Middle (4–7)		2343 (38·9%)	1559 (39·7%)	784 (37·4%)	899 (37·9%)	517 (38·7%)	382 (36·9%)	2283 (37·9%); p=0·27	1624 (38·7%); p=0·39	659 (35·9%); p=0·37	915 (38·6%); p=0·72	581 (38·8%); p=0·25	334 (38·3%); p=0·81
High (8–10)		2471 (41·0%)	1553 (39·5%)	918 (43·7%)	1035 (43·7%)	558 (41·8%)	477 (46·1%)	2694 (44·7%); p<0·0001	1779 (42·4%); p=0·0082	915 (49·9%); p=0·0001	1062 (44·8%); p=0·39	631 (42·1%); p=0·47	431 (49·5%); p=0·12
**Healthy lifestyle**†													
No current smoking		3429/3519 (97·4%)	2265/2337 (96·9%)	1164/1182 (98·5%)	1431/1455 (98·4%)	828/844 (98·1%)	603/611 (98·7%)	3448/3550 (97·1%); p=0·42	2461/2549 (96·5%); p=0·47	987/1001 (98·6%); p=0·85	1431/1468 (97·5%); p=0·12	925/956 (96·8%); p=0·077	506/512 (98·8%); p=1·0
No obesity		2654/3519 (75·4%)	1756/2337 (75·1%)	898/1182 (76·0%)	1096/1455 (75·3%)	615/844 (72·9%)	481/611 (78·7%)	2787/3550 (78·5%); p=0·0027	1967/2549 (77·2%); p=0·11	820/1001 (81·9%); p=0·0008	1141/1468 (77·7%); p=0·13	723/956 (75·6%); p=0·16	418/512 (81·6%); p=0·32
Healthier diet		1284/3519 (36·5%)	756/2337 (32·3%)	528/1182 (44·7%)	575/1455 (39·5%)	278/844 (32·9%)	297/611 (48·6%)	1372/3550 (38·6%); p=0·062	887/2549 (34·8%); p=0·070	485/1001 (48·5%); p=0·085	625/1468 (42·6%); p=0·098	370/956 (38·7%); p=0·012	255/512 (49·8%); p=0·72
Not sedentary		2845/3519 (80·8%)	1903/2337 (81·4%)	942/1182 (79·7%)	1196/1455 (82·2%)	679/844 (80·5%)	517/611 (84·6%)	2831/3550 (79·7%); p=0·24	2033/2549 (79·8%); p=0·13	798/1001 (79·7%); p=0·96	1180/1468 (80·4%); p=0·22	768/956 (80·3%); p=0·95	412/512 (80·5%); p=0·023
Healthy lifestyle score		2·9 (0·8)	2·9 (0·8)	3·0 (0·9)	3·0 (0·8)	2·9 (0·8)	3·1 (0·8)	2·9 (0·9); p=0·021	2·9 (0·9); p=0·17	3·1 (0·8); p=0·078	3·0 (0·9); p=0·11	2·9 (0·9); p=0·018	3·1 (0·8); p=0·44

Data are n (%), n (%); p value, n/N (%), n/N (%); p value, or mean (SD). The p values indicate the difference between cases 1 and controls 1, or between cases 2 and controls 2, and were not adjusted for multiple testing.

* Insufficient sample size to calculate p value.

† We only included those who answered the relevant questionnaire in the healthy lifestyle factors analysis.

Asthma and lung disease were the most commonly reported comorbidities (table 1). There was a significant difference in the prevalence of several individual comorbidities between groups, including cancer (more prevalent in older adults in the control groups than in the case groups), diabetes (more prevalent in younger adults in controls 1 than in cases 1), lung disease (more prevalent in older adults in cases 1 than in controls 1), and asthma (more prevalent in older adults in cases 1 than in controls 1 and more prevalent in cases 2 than in controls 2; table 1). There were also significant differences in vaccine type between groups, with significantly higher proportions of cases than controls receiving ChAdOx1 nCoV-19, and significantly higher proportions of controls than cases receiving BNT162b2 across all groups (table 1). Only 86 of 17 958 individuals and one of 3417 individuals reported SARS-CoV-2 infection following their first and second dose of the mRNA-1273 vaccine in this study, respectively.

**Figure 1:**
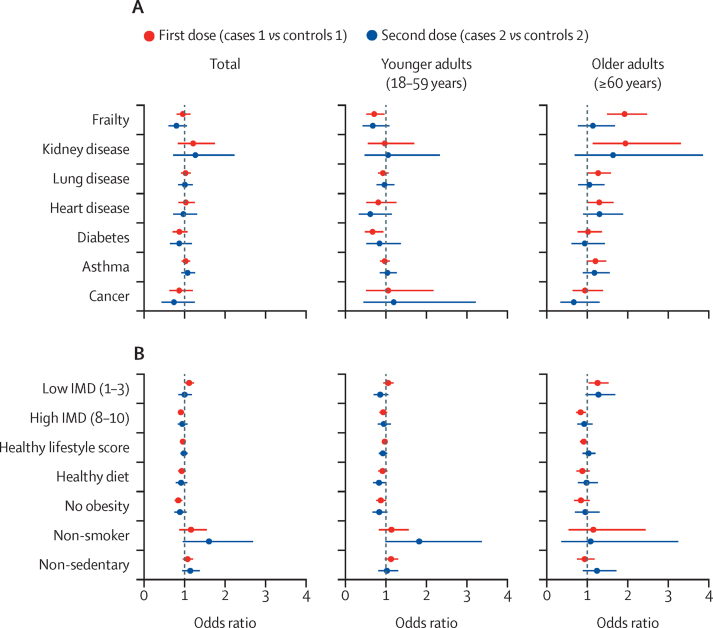
Univariate analysis of post-vaccination SARS-CoV-2 infection risk factors Univariate models for frailty and each individual comorbidity (A) and IMD, healthy lifestyle factors, and healthy lifestyle score (B), adjusted for age, body-mass index, and sex, and stratified by age group. The error bars represent 95% CIs. A low IMD means high deprivation and a high IMD means low deprivation. The reference category for the IMD is an intermediate IMD (4–7). IMD=Index of Multiple Deprivation.

In the multivariate analysis of age and BMI, adjusted for sex, we found a significant inverse association between age and post-vaccination infection that was more evident in older adults after the first dose (OR 0·94 per year increase in age, 95% CI 0·93–0·95; p<0·0001) and after the second (0·93, 0·92–0·95; p<0·0001) than in younger adults (appendix p 2). In univariate logistic regression models adjusted for age, BMI, and sex, frailty was associated with post-vaccination infection in older adults following their first vaccine dose (OR 1·93, 95% CI 1·50–2·48; p<0·0001; figure 1A; appendix p 2), an association that remained consistent in our sensitivity analysis using inverse probability weighting for factors influencing vaccination (appendix p 3). 30 (23%) of 130 frail older adults presented to hospital after testing positive for SARS-CoV-2 following their first vaccine dose and two (6%) of 36 frail older adults presented to hospital after testing positive for SARS-CoV-2 following their second vaccine dose. In older adults who had received their first vaccine dose but not their second, kidney disease (OR 1·95, 95% CI 1·14–3·31; p=0·014), heart disease (1·30, 1·03–1·65; p=0·031), and lung disease (1·27, 1·02–1·59; p=0·030) were associated with post-vaccination infection (appendix p 2; figure 1A).

Full results of our sensitivity analysis using inverse probability weighting for factors influencing vaccination (frailty, each comorbidity, and IMD categories) can be found in the appendix (pp 3–4). Generally, our sensitivity analysis confirmed the results of our main analysis; however, beyond frailty, older adults with asthma and lung disease had a significantly increased odds of infection after their first vaccine dose in our sensitivity analysis (appendix p 3). Furthermore, a significantly decreased odds of post-vaccination infection were found in younger adults with frailty following their first and second vaccine doses, in younger adults with heart disease following their second vaccine dose, and in younger adults with diabetes following their first vaccine dose (appendix p 3).

Compared with the intermediate IMD category, users living in areas with the highest deprivation (a low IMD of 1–3) had increased odds of SARS-CoV-2 infection following their first vaccine dose (OR 1·11, 95% CI 1·01–1·23; p=0·039), and users living in areas with the lowest deprivation (a high IMD of 8–10) had decreased odds of SARS-CoV-2 infection following their first vaccine dose (0·91, 0·84–0·98; p=0·017; figure 1B; appendix p 3). Individuals without obesity had decreased odds of infection following their first vaccine dose, which was seen in the main analysis in all age groups (OR 0·84, 95% CI 0·75–0·94; p=0·0030; figure 1B; appendix p 3) and in older adults in the inverse probability weighting sensitivity analysis (appendix p 4). The results of our univariate analysis remained similar after adjusting for adherence to mask wearing (appendix p 16).

**Figure 2:**
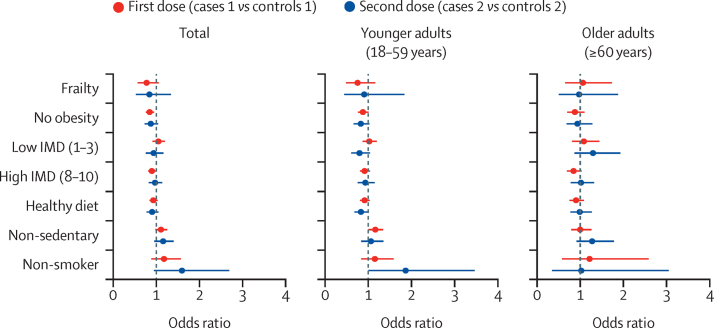
Multivariate analysis of post-vaccination SARS-CoV-2 infection risk factors The multivariate analysis was adjusted for age, body-mass index, and sex, and was stratified by age group. The error bars represent 95% CIs. A low IMD means high deprivation and a high IMD means low deprivation. The reference category for the IMD is an intermediate IMD (4–7). IMD=Index of Multiple Deprivation.

In multivariate analyses adjusted by age, BMI, and sex (figure 2), lower deprivation (for all ages) and non-obesity (for younger adults and all ages) were independently associated with post-vaccination infection following the first vaccine dose (appendix p 5). However, frailty was not significantly associated with post-vaccination infection after the first or second dose in any age group (figure 2; appendix p 5). The findings of the multivariate analysis were broadly consistent following our sensitivity analysis using inverse probability weighting for factors influencing vaccination (appendix p 6).

For our analysis of symptom duration and disease severity, we used data collected up to July 18, 2021, to ensure that all participants had at least 28 days post-vaccination for symptom reporting. Among the 6030 app users in cases 1, 3825 had at least 14 days of app use after testing positive for SARS-CoV-2 (cases 3; median duration of app use 79 days [IQR 30–135]), and, among the 2370 app users in cases 2, 906 had at least 14 days of app use after testing positive for SARS-CoV-2 (cases 4; median duration of app use 27 days [IQR 20–45]). After the first vaccine dose, positive tests were by RT-PCR in 70·0% of cases and in 49·2% of controls, although 41·3% of controls did not report on the type of test done (appendix p 6). After the second vaccine dose, the proportion of tests done by RT-PCR was similar for both cases and controls (appendix p 6).

**Figure 3:**
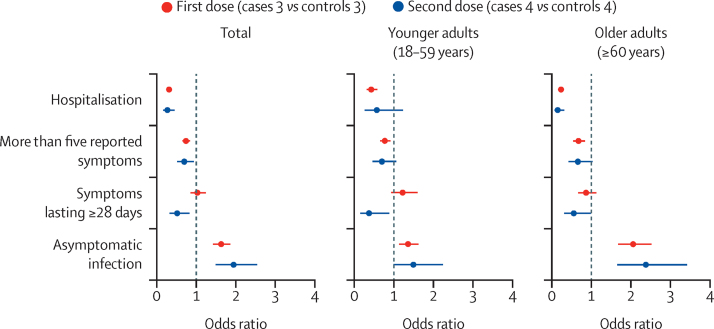
Disease severity and duration factors in SARS-CoV-2-infected vaccinated versus unvaccinated participants Univariate models were adjusted for age, body-mass index, and sex, and stratified by age group. The error bars represent 95% CIs.

Following matching, there was still a significantly higher prevalence of health-care workers in cases 3 (compared with controls 3) and cases 4 (compared with controls 4; table 2). In univariate models adjusted by age, BMI, and sex, there were lower odds of long-duration (≥28 days) symptoms following two vaccine doses for all participants (OR 0·51, 95% CI 0·32–0·82; p=0·0060; appendix p 7). Compared with unvaccinated controls, individuals after their first or second vaccine dose were less likely to have more than five symptoms in the first week of illness or present to hospital, and were more likely to be completely asymptomatic, especially if they were 60 years or older (figure 3; appendix p 7).

**Table 2:** Characteristics of case participants with COVID-19 after their first or second vaccination and unvaccinated control participants with COVID-19 in the disease profile analysis

		**Cases 3**			**Cases 4**			**Controls 3**			**Controls 4**		
		Total (n=3825)	18–59 years (n=2320)	≥60 years (n=1505)	Total (n=906)	18–59 years (n=455)	≥60 years (n=451)	Total (n=3825)	18–59 years (n=2363)	≥60 years (n=1462)	Total (n=906)	18–59 years (n=474)	≥60 years (n=432)
**Demographics**													
Sex													
	Female	2460 (64·3%)	1578 (68·0%)	882 (58·6%)	561 (61·9%)	305 (67·0%)	256 (56·8%)	2462 (64·4%); p=0·98	1590 (67·3%); p=0·60	872 (59·6%); p=0·58	553 (61·0%); p=0·74	296 (62·4%); p=0·15	257 (59·5%); p=0·41
	Male	1365 (35·7%)	742 (32·0%)	623 (41·4%)	345 (38·1%)	150 (33·0%)	195 (43·2%)	1363 (35·6%)	773 (32·7%)	590 (40·4%)	353 (39·0%)	178 (37·6%)	175 (40·5%)
Age, years		52·0 (14·2)	43·2 (9·0)	65·6 (9·0)	54·5 (14·3)	43·4 (9·6)	65·7 (8·4)	51·5 (14·2); p=0·20	42·9 (9·3); p=0·25	65·4 (8·8); p=0·22	53·7 (13·8); p=0·55	43·6 (9·6); p=0·36	64·9 (7·9); p=0·084
Body-mass index, kg/m^2^		27·2 (6·8)	27·3 (6·9)	27·1 (6·7)	26·9 (7·3)	27·3 (7·7)	26·5 (7·0)	27·2 (6·6); p=0·71	27·3 (6·8); p=0·44	27·1 (6·2); p=0·28	26·9 (6·7); p=0·83	27·2 (7·0); p=0·45	26·6 (6·2); p=0·19
Health-care worker		683 (17·9%)	475 (20·5%)	208 (13·8%)	124 (13·7%)	90 (19·8%)	34 (7·5%)	572 (15·0%); p=0·0006	393 (16·6%); p=0·0008	179 (12·2%); p=0·21	91 (10·0%); p=0·020	58 (12·2%); p=0·0022	33 (7·6%); p=1·0
**Comorbidities**													
Frailty		215 (5·6%)	49 (2·1%)	166 (11·0%)	51 (5·6%)	14 (3·1%)	37 (8·2%)	138 (3·6%); p<0·0001	24 (1·0%); p=0·0029	114 (7·8%); p=0·0031	21 (2·3%); p=0·0004	4 (0·8%); p=0·016	17 (3·9%); p=0·018
Presence of at least one comorbidity		891 (23·3%)	453 (19·5%)	438 (29·1%)	236 (26·0%)	111 (24·4%)	125 (27·7%)	809 (21·2%); p=0·026	391 (16·5%); p=0·0087	418 (28·6%); p=0·78	207 (22·8%); p=0·13	76 (16·0%); p=0·0018	131 (30·3%); p=0·41
Disease characteristics*													
Asymptomatic infection		672/3683 (18·2%)	331/2234 (14·8%)	341/1449 (23·5%)	183/887 (20·6%)	66/444 (14·9%)	117/443 (26·4%)	414/3445 (12·0%); p<0·0001	242/2121 (11·4%); p=0·0009	172/1324 (13·0%); p<0·0001	98/835 (11·7%); p<0·0001	45/425 (10·6%); p=0·067	53/410 (12·9%); p<0·0001
Hospitalised		147/3791 (3·9%)	64/2298 (2·8%)	83/1493 (5·6%)	20/897 (2·2%)	11/451 (2·4%)	9/446 (2·0%)	397/3726 (10·7%); p<0·0001	142/2297 (6·2%); p<0·0001	255/1429 (17·8%); p<0·0001	64/867 (7·4%); p<0·0001	18/445 (4·0%); p=0·19	46/422 (10·9%); p<0·0001
More than five reported symptoms		551/2479 (22·2%)	368/1430 (25·7%)	183/1049 (17·4%)	121/592 (20·4%)	71/286 (24·8%)	50/306 (16·3%)	868/2762 (31·4%); p<0·0001	515/1540 (33·4%); p<0·0001	353/1222 (28·9%); p<0·0001	141/482 (29·3%); p=0·0010	71/223 (31·8%); p=0·090	70/259 (27·0%); p=0·0027
Symptoms lasting ≥28 days		229/2479 (9·2%)	124/1430 (8·7%)	105/1049 (10·0%)	31/592 (5·2%)	9/286 (3·1%)	22/306 (7·2%)	296/2762 (10·7%); p=0·080	121/1540 (7·9%); p=0·42	175/1222 (14·3%); p=0·0020	55/482 (11·4%); p=0·0002	16/223 (7·2%); p=0·040	39/259 (15·1%); p=0·0040
**Reporting**													
Duration of reporting, days		180·7 (51·5)	174·4 (55·6)	191·4 (41·5)	192·1 (42·7)	184·5 (49·9)	201·0 (30·0)	155·7 (64·6); p<0·0001	150·4 (65·8); p<0·0001	164·4 (61·7); p<0·0001	178·7 (51·0); p<0·0001	171·3 (55·9); p=0·0006	186·7 (43·7); p<0·0001
Proportion of missing daily reports, %		50% (30)	60% (30)	50% (20)	50% (30)	60% (30)	40% (20)	60% (30); p<0·0001	70% (30); p<0·0001	50% (30); p=0·13	60% (30); p<0·0001	70% (30); p=0·0011	50% (30); p=0·48

Data are n (%), n (%); p value, n/N (%), n/N (%); p value, or mean (SD). The p values indicate the difference between cases 3 and controls 3, or between cases 4 and controls 4, and were not adjusted for multiple testing.

* Only users with information on hospitalisation, symptoms, and disease duration were included in the analysis.

**Figure 4:**
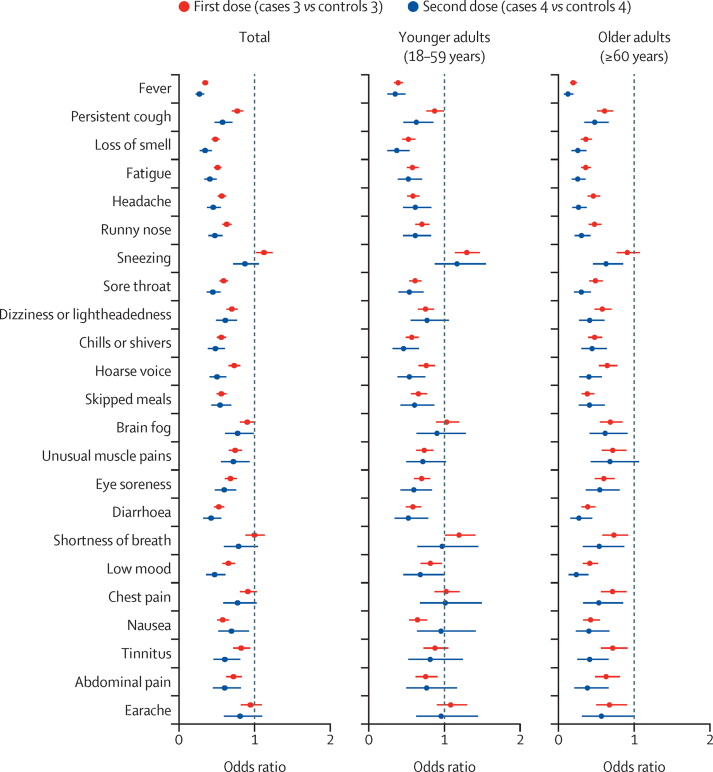
Symptoms in SARS-CoV-2-infected vaccinated versus unvaccinated participants Univariate models were adjusted for age, body-mass index, and sex, and stratified by age group. The error bars represent 95% CIs. We present only symptoms reported by more than 1% of users.

For our symptom reports, we used data collected up to July 4, 2021. Symptom frequencies for participants testing positive for SARS-CoV-2 after their first or second vaccination and for unvaccinated control participants can be found in the appendix (pp 7–9). Vaccination was associated with lower symptom reporting for almost all symptoms across all age groups (figure 4; appendix pp 9–10). One exception was sneezing (sternutation), which was more common in vaccinated individuals than in unvaccinated controls after the first vaccine dose, although only when considering all age groups and younger adults (appendix pp 9–10). When considering all age groups, no differences were found between cases and controls for chest pain, lymphadenopathy (swollen glands), and earache following first or second vaccine doses; dyspnoea (shortness of breath) following the second dose; brain fog following the first dose; or sneezing following the second dose. Results from the univariate analysis remained similar after further adjustment for frailty and the presence of at least one comorbidity (appendix pp 11–13).

## Discussion

We present data on 6030 and 2370 community-based adults in the UK with test-confirmed SARS-CoV-2 infection after their first or second COVID-19 vaccinations, respectively, with BNT162b2, ChAdOx1 nCoV-19, or mRNA-1273. Participants were included if they tested positive for SARS-CoV-2 at least 14 days after their first vaccination or at least 7 days after their second vaccination when immunity had developed^32^ and infection was unlikely to be due to exposure around the time of vaccination (eg, when travelling to the vaccination centre).

We found that the odds of having symptoms for 28 days or more after post-vaccination infection were approximately halved by having two vaccine doses. This result suggests that the risk of long COVID is reduced in individuals who have received double vaccination, when additionally considering the already documented reduced risk of infection overall.^2^, ^3^, ^4^, ^6^, ^7^, ^8^

Almost all individual symptoms of COVID-19 were less common in vaccinated versus unvaccinated participants, and more people in the vaccinated than in the unvaccinated groups were completely asymptomatic. This increased incidence of asymptomatic or minimally symptomatic infection in vaccinated participants underlines the importance of individuals who interact with unvaccinated or clinically vulnerable groups (eg, health-care workers and social care workers) continuing to regularly take tests for SARS-CoV-2, even if vaccinated, in line with current UK testing guidelines.^33^ We also found that COVID-19 was less severe (both in terms of the number of symptoms in the first week of infection and the need for hospitalisation) in participants after their first or second vaccine doses compared with unvaccinated participants. We have previously shown that having more than five symptoms in the first week of infection was associated with COVID-19 severity^20^ and disease duration.^21^

Frailty was associated with post-vaccination infection in older adults following their first vaccine dose, highlighting the need for ongoing caution in this clinically vulnerable group. The association was consistent in our sensitivity analysis using inverse probability weighting for factors influencing vaccination, but not after adjusting for potential confounders such as local area deprivation and lifestyle. This increased risk might therefore reflect increased exposure: unlike non-frail older adults, frail older adults might require carer visits or attendance at health-care facilities. Frail adults in long-term care facilities are at particular risk of transmitting respiratory illness, and have been disproportionately affected throughout the COVID-19 pandemic.^17^ Another explanation for this result concerns altered immune function (immunosenescence), a well established feature of physiological ageing.^34^, ^35^ The increased odds of post-vaccination infection in frail older adults could be compounded by the more severe outcomes of COVID-19 in this group, including delirium^25^ and death;^17^ indeed, in our study, 23% of frail, older adults testing positive for SARS-CoV-2 after their first vaccination presented to hospital. Systematic frailty screening in acute and community-based settings might facilitate differential, targeted re-vaccination scheduling, appropriate isolation precautions, case detection, testing, and proactive care, as recommended in guidance published by the National Institute for Health and Care Excellence^36^ and National Health Service (NHS) England.^37^ Research on augmenting immunogenicity in frail individuals is needed, such as on the impact and timing of booster vaccinations.

We found an inverse association of age with the odds of post-vaccination infection, especially in older adults. This finding is consistent with a previous study in non-vaccinated individuals showing lower anti-SARS-CoV-2 antibody seroprevalence in older adults (≥65 years) compared with younger adults (35–44 years),^38^ perhaps reflecting shielding in this age group in accordance with the classification of individuals older than 70 years as clinically vulnerable. Our study found some evidence to suggest that kidney disease might increase the odds of SARS-CoV-2 infection in older adults after their first vaccine dose, which is notable given that individuals with kidney disease were under-represented in the phase 2 and phase 3 trials of the COVID-19 vaccines.^39^ However, this finding should be interpreted cautiously because of the relatively small numbers of participants with kidney disease in this study. This increased risk of post-vaccination infection for people with kidney disease could reflect increased exposure (eg, when attending dialysis appointments) or impaired immunogenicity, and is supported by a study looking at humoral and B-cell responses in vaccinated, immunosuppressed kidney transplant recipients and patients having dialysis.^40^ Several other comorbidities, including heart disease and lung disease, were significantly associated with post-vaccination infection after one dose in older adults; although associations of marginal significance should be interpreted cautiously, many of these comorbidities confer a higher risk of severe disease, hospitalisation, mechanical ventilation, and mortality from COVID-19,^16^, ^18^ and ongoing shielding behaviours could be influencing our results to reduce the strength of these associations.

Greater area-level deprivation was associated with increased odds of SARS-CoV-2 infection after a single vaccine dose, consistent with findings from the pre-vaccination era.^15^ This association persisted following further adjustment for compliance with infection control guidance (ie, mask wearing). Factors associated with increased area-level deprivation include higher population density and more ethnic diversity, which are in themselves associated with increased mortality from COVID-19.^18^ More deprived areas might have lower vaccination coverage for COVID-19,^41^ and our finding might reflect increased viral transmission. Our results suggest that health policies to mitigate infection might need to specifically target these areas. Conversely, individuals without obesity had a lower odds of infection following their first vaccine dose. This finding suggests that immune responses post-vaccination might be influenced by obesity, although unadjusted confounding remains a possibility.

Our observation of differences by vaccine type agrees with real-world UK data on the effectiveness of ChAdOx1 nCoV-19 and BNT162b2 against the delta variant (B.1.617.2);^42^, ^43^ however, our observation should be treated cautiously given the confounding factors influencing the vaccine type administered in different age groups and demographics. We emphasise that our study is observational, rather than a formal comparison.

Our study has some limitations. Although we used data from a large population of individuals reporting on a mobile phone app, the sample contained disproportionately more women than men and under-represented individuals in more deprived areas. Furthermore, we were unable to analyse the impact of ethnicity due to the low number of participants who provided this information, and our findings might not apply at all timepoints post-vaccination, to settings with different proportions of SARS-CoV-2 variants, or to countries with a different vaccination schedule. Additionally, the data were self-reported; recording of comorbidities, test results, and vaccination status might not have been completely accurate and there might have been temporal gaps in reporting. Users of the COVID Symptom Study app are asked to log daily; therefore, if a participant reports on alternate days, the proportion of missing daily entries is 50%. However, given the typical duration of COVID-19 symptoms, the sampling frequencies in the COVID Symptom Study should have allowed good characterisation of infections.

Our study has strengths. Previous data from the COVID Symptom Study have concurred well with population-based COVID-19 studies,^44^ including on the influence of sociodemographic factors.^15^ The mobile phone data collection method allows the collection of daily prospective information on a comprehensive set of symptoms, permitting the analysis of both individual symptoms and overall illness duration (although necessary data censoring could have underestimated symptom duration in both cases and controls, as some individuals only had 2 weeks of logging after their positive test result).

The design of our study, including matching cases and controls for health-care worker status and the date of the post-vaccination test, reduced the potential for bias, although small differences between the groups remained on matched variables. We acknowledge the potential differences in logging by vaccinated individuals or those undertaking regular testing (eg, required for work as a health-care worker). Access to testing is a potential source of bias: as of July, 2021, RT-PCR is recommended for symptomatic individuals and a LFAT is recommended for asymptomatic individuals in the UK.^33^ The risk of reporting a positive SARS-CoV-2 test is higher among frontline health-care workers than among the general population,^14^ probably reflecting increased exposure and testing. After the first vaccine dose, positive tests were by RT-PCR in a higher proportion of vaccinated cases than in unvaccinated controls. Because RT-PCR is used for symptomatic testing in the UK, this would bias away our finding of a higher likelihood of asymptomatic or minimally symptomatic COVID-19 in vaccinated versus unvaccinated participants. However, there is uncertainty due to the high numbers of unvaccinated controls who reported an unknown test type after their first vaccine dose.

Our data suggest that the risk of post-vaccination SARS-CoV-2 infection is reduced in older age groups. To examine the effect of age on post-vaccination infection, we did not match controls 1 and controls 2 by age. However, age was included as a covariate in all analyses other than that looking at the effect of age itself, and stratified analyses are presented for younger and older age groups. Although vaccination itself might be considered a potential index event bias, the population of interest in this study was the vaccinated population, and findings should not be construed as applying to those who are unvaccinated. The UK adopted a vaccine prioritisation strategy starting with older age groups, so older adults were more likely to be vaccinated in this study than were younger adults.^45^ We examined and found no evidence of event bias on the basis of the probability of being vaccinated. Analyses in this study were not corrected for multiple testing, and so observations of marginal significance should be interpreted cautiously.

Frailty was assessed with the PRISMA-7 questionnaire. This assessment correlates well with other frailty measures^46^ and has the advantage of focusing on the functional consequences of frailty, which are not routinely captured in health records. However, PRISMA-7 has only been validated in older adults; results in younger adults should be interpreted cautiously.^24^

To conclude, the odds of post-vaccination infection following the first dose were increased in frail, older adults and in those living in more deprived areas, and were decreased in individuals without obesity. Compared with unvaccinated controls, after their second vaccine dose, individuals were less likely to have prolonged illness (symptoms for ≥28 days), more than five symptoms in the first week of illness, or present to hospital. Most symptoms were less common in vaccinated versus unvaccinated participants. Fully vaccinated individuals with COVID-19, especially if they were 60 years or older, were more likely to be completely asymptomatic than were unvaccinated controls. This finding might support caution around relaxing physical distancing and other personal protective measures in the post-vaccination era, particularly around frail older adults and individuals living in more deprived areas, even if these individuals are vaccinated. Our findings might also have implications for strategies such as booster vaccinations.

## Data sharing

Deidentified participant data collected in the COVID Symptom Study smartphone app can be shared with other health researchers through the NHS-funded Health Data Research UK and Secure Anonymised Information Linkage consortium, housed in the UK Secure Research Platform (Swansea, UK). Anonymised data are available to be shared with researchers according to their protocols in the public interest (https://web.www.healthdatagateway.org/dataset/fddcb382-3051-4394-8436-b92295f14259). Researchers must apply to gain access through Health Data Research UK.

## Declaration of interests
