# Supplementary material for: Risk factors and disease profile of post-vaccination SARS-CoV-2 infection in UK users of the COVID Symptom Study app: a prospective, community-based, nested, case-control study
Source: Lancet Infect Dis. 2022 Jan;22(1):43–55. doi: 10.1016/S1473-3099(21)00460-6 (PMC8409907; doi:10.1016/S1473-3099(21)00460-6)
Supplement: Supplementary appendix [file mmc1.pdf]

THE LANCET
Infectious Diseases

Supplementary appendix

This appendix formed part of the original submission and has been peer reviewed. We post it as supplied by the authors.

Supplement to: Antonelli M, Penfold RS, Merino J. Risk factors and disease profile of post-vaccination SARS-CoV-2 infection in UK users of the COVID Symptom Study app: a prospective, community-based, nested, case-control study. *Lancet Infect Dis* 2021; published online September 1. https://doi.org/10.1016/S1473-3099(21)00460-6.

Supplementary Table 1. List of self-reported symptoms and corresponding questions used in the reporting app.

Symptoms	Questions
Fever	Do you have a fever or feel too hot?
Chills or shivers	Do you feel chills or shivers (feel too cold)?
Persistent cough	Persistent cough (coughing a lot for more than an hour or 3 or more coughing episodes in 24 hours)
Fatigue	Are you experiencing unusual fatigue?
Shortness of breath	Shortness of breath or trouble breathing
Loss of smell	Loss of smell / taste
Hoarse voice	Unusually hoarse voice
Chest pain	Unusual chest pain or tightness in your chest
Abdominal pain	Unusual abdominal pain or stomach ache
Diarrhoea	Diarrhoea
Delirium or reported confusion	Confusion, disorientation or drowsiness
Eye soreness	Do your eyes have any unusual eye-soreness or discomfort (e.g. light sensitivity, excessive tears, or pink/red eye)?
Skipped meals	Skipping meals
Headache	Headache
Nausea	Nausea or vomiting
Dizziness/ lightheadedness	Dizziness or light-headedness
Sore throat	Sore or painful throat
Unusual muscle pains	Unusual strong muscle pains or aches
Red welts on face or lips	Raised, red, itchy welts on the skin or sudden swelling of the face or lips
Blisters on feet	Red/purple sores or blisters on your feet, including your toes
Typical hayfever	Increase in your usual allergy symptoms
Rash	Rash on your arms or torso
Sensation of skin burning	Strange, unpleasant sensations in your skin like “pins & needles” or burning
Hair loss	Unusual hair loss
Low mood	Feeling down, depressed or hopeless
Brain fog	Loss of concentration or memory (brain fog)
Runny nose	Runny nose
Sneezing	Sneezing more than usual
Earache	Earache
Tinnitus	Ringing in your ears
Swollen glands	Swollen neck glands
Irregular heartbeat	Unusually fast or irregular heartbeat (palpitations)

Supplementary Table 2. Number and proportion of type of tests performed by cases testing positive and controls testing negatives after the first and second dose

	**Cases-1-Cases-2 n=6030 (D1) n=2370 (D2)**			**Controls-1-Controls-2 n=6030 (D1) n=2370 (D2)**		
	RT-PCR confirmed tests n(%)	LFAT confirmed tests n(%)	Not sure n(%)	RT-PCR confirmed tests n(%)	LFAT confirmed tests n(%)	Not sure n(%)
D1	4,200(70%)	1,583(26%)	247(4%)	4,556(76%)	1,391(23%)	83(1%)
D2	1,570(66%)	746(31%)	54(3%)	2,020(85%)	338(14%)	12(1%)

n=number of individuals; D1=After first dose; D2=After second dose; Cases-1=cases infected after the first dose but before the second dose of the vaccine; Cases-2=cases infected after the second dose of the vaccine; Controls-1 and Controls-2=vaccinated users tested negative after first and second dose matched with Cases-1 and Cases-2, respectively.

Supplementary Table 3. Multivariate analysis of age and body mass index (BMI), adjusted by sex.

		All age groups			Younger adults (18-59 years)			Older adults (60+ years)		
		OR	95% CI	p-value	OR	95% CI	p-value	OR	95% CI	p-value
BMI	D1	1·00	[1·00-1·01]	0·023	1·00	[1·00-1·01]	0·22	1·01	[1·00-1·02]	0·037
	D2	1·00	[0·99-1·00]	0·81	1·00	[0·99-1·01]	0·88	1·00	[0·99-1·02]	0·69
Age (years)	D1	0·99	[0·99- 1·00]	<0·0001	0·98	[0·98-0·98]	<0·0001	0·94	[0·93-0·95]	<0·0001
	D2	0·99	[0·99-0·99]	<0·0001	0·97	[0·96-0·98]	<0·0001	0·93	[0·92-0·95]	<0·0001

OR = Odds ratio; CI = Confidence interval; BMI = Body mass index; D1=After first dose; D2=After second dose

Supplementary Table 4. Univariate analysis of frailty status and each comorbidity, adjusted by age, BMI, and sex.

		All age groups			Younger adults (18-59 years)			Older adults (60+ years)		
		OR	95% CI	p-value	OR	95% CI	p-value	OR	95% CI	p-value
frailty	D1	0·95	[0·80-1·14]	0·61	0·71	[0·52-0·97]	0·029	1·93	[1·50-2·48]	<0·0001
	D2	0·80	[0·60-1·06]	0·12	0·68	[0·42-1·09]	0·106	1·14	[0·78-1·69]	0·50
kidney disease	D1	1·21	[0·84-1·75]	0·31	0·97	[0·55-1·70]	0·917	1·95	[1·14-3·31]	0·014
	D2	1·27	[0·72-2·23]	0·42	1·05	[0·48-2·33]	0·898	1·64	[0·70-3·86]	0·26
lung disease	D1	1·02	[0·91-1·15]	0·72	0·93	[0·81-1·07]	0·294	1·27	[1·02-1·59]	0·030
	D2	1·01	[0·84-1·20]	0·96	0·97	[0·77-1·21]	0·765	1·06	[0·78-1·43]	0·71
heart disease	D1	1·03	[0·84-1·25]	0·78	0·81	[0·52-1·26]	0·354	1·30	[1·03-1·65]	0·031
	D2	0·97	[0·72-1·31]	0·83	0·61	[0·33-1·14]	0·124	1·31	[0·90-1·89]	0·16
diabetes	D1	0·87	[0·71-1·07]	0·19	0·67	[0·48-0·94]	0·019	1·02	[0·77-1·36]	0·87
	D2	0·87	[0·64-1·18]	0·36	0·84	[0·52-1·37]	0·487	0·94	[0·62-1·44]	0·79
asthma	D1	1·02	[0·92-1·14]	0·640	0·97	[0·86-1·10]	0·635	1·21	[0·99-1·47]	0·063
	D2	1·07	[0·92-1·26]	0·38	1·04	[0·85-1·26]	0·718	1·18	[0·90-1·56]	0·23
cancer	D1	0·87	[0·62-1·20]	0·39	1·06	[0·51-2·17]	0·880	0·95	[0·65-1·40]	0·79
	D2	0·74	[0·43-1·25]	0·26	1·19	[0·44-3·23]	0·726	0·67	[0·35-1·30]	0·24

OR = Odds ratio; CI = Confidence interval; BMI = Body mass index; D1=After first dose; D2=After second dose

Supplementary Table 5. Sensitivity analysis of frailty and each comorbidity using Inverse Probability Weighting (IPW) for probability of vaccination. Each univariate analysis is adjusted by age, BMI, and sex.

		All age groups			Younger adults (18-59 years)			Older adults (60+ years)		
		OR	95% CI	p-value	OR	95% CI	p-value	OR	95% CI	p-value
frailty	D1	0·96	[0·81-1·12]	0·57	0·69	[0·53-0·90]	0·007	2·08	[1·65-2·62]	<0·0001
	D2	0·79	[0·61-1·01]	0·07	0·63	[0·42-0·96]	0·032	1·19	[0·84-1·68]	0·34
kidney disease	D1	1·12	[0·80-1·58]	0·51	0·89	[0·53-1·48]	0·649	1·8	[1·10-2·96]	0·020
	D2	1·34	[0·80-2·24]	0·27	1·03	[0·50-2·12]	0·943	1·9	[0·86-4·22]	0·11
lung disease	D1	1·02	[0·92-1·13]	0·69	0·9	[0·80-1·02]	0·104	1·38	[1·13-1·67]	0·0010
	D2	1·01	[0·87-1·18]	0·86	0·95	[0·78-1·15]	0·579	1·16	[0·89-1·52]	0·27
heart disease	D1	1·01	[0·83-1·21]	0·96	0·78	[0·52-1·19]	0·247	1·29	[1·03-1·61]	0·028
	D2	0·9	[0·68-1·20]	0·48	0·56	[0·31-0·99]	0·045	1·25	[0·88-1·76]	0·22
diabetes	D1	0·78	[0·64-0·94]	0·010	0·62	[0·46-0·84]	0·002	0·88	[0·67-1·14]	0·33
	D2	0·74	[0·56-0·99]	0·041	0·72	[0·46-1·12]	0·143	0·78	[0·53-1·16]	0·22
asthma	D1	1·02	[0·93-1·11]	0·70	0·96	[0·87-1·07]	0·448	1·23	[1·04-1·47]	0·019
	D2	1·07	[0·93-1·23]	0·34	1·02	[0·86-1·21]	0·81	1·25	[0·98-1·59]	0·073
cancer	D1	0·78	[0·57-1·08]	0·14	1·21	[0·62-2·35]	0·574	0·8	[0·54-1·17]	0·24
	D2	0·72	[0·44-1·19]	0·20	1·34	[0·54-3·35]	0·532	0·61	[0·32-1·14]	0·12
OR = Odds ratio; CI = Confidence interval; BMI = Body mass index; D1=After first dose; D2=After second dose										

Supplementary Table 6. Univariate analysis of Index of Multiple Deprivation (IMD) category, obesity status and healthy lifestyle factors.

		All age groups			Younger adults (18-59 years)			Older adults (60+ years)		
		OR	95% CI	p-value	OR	95% CI	p-value	OR	95% CI	p-value
IMD[1-3]	D1	1·11	[1·01-1·23]	0·039	1·06	[0·94-1·19]	0·383	1·26	[1·03-1·53]	0·021
	D2	1·00	[0·85-1·18]	0·98	0·86	[0·70-1·06]	0·161	1·28	[0·96-1·69]	0·091
IMD[8-10]	D1	0·91	[0·84-0·98]	0·017	0·93	[0·85-1·03]	0·174	0·84	[0·73-0·96]	0·014
	D2	0·94	[0·83-1·07]	0·37	0·95	[0·80-1·13]	0·554	0·93	[0·76-1·13]	0·46
Healthy lifestyle score	D1	0·96	[0·91-1·01]	0·12	0·97	[0·91-1·04]	0·443	0·91	[0·82-1·02]	0·095
	D2	0·98	[0·90-1·07]	0·67	0·93	[0·83-1·03]	0·174	1·03	[0·89-1·20]	0·68
Healthier diet	D1	0·93	[0·84-1·03]	0·14	0·92	[0·82-1·04]	0·176	0·88	[0·73-1·06]	0·18
	D2	0·91	[0·79-1·06]	0·23	0·83	[0·68-1·01]	0·066	0·98	[0·77-1·26]	0·89
Non obese	D1	0·84	[0·75-0·94]	0·0030	0·88	[0·77-1·00]	0·052	0·85	[0·67-1·06]	0·15
	D2	0·88	[0·74-1·05]	0·17	0·84	[0·67-1·04]	0·105	0·95	[0·70-1·30]	0·77
Non smoker	D1	1·16	[0·87-1·54]	0·32	1·14	[0·83-1·57]	0·422	1·15	[0·54-2·44]	0·71
	D2	1·60	[0·95-2·70]	0·075	1·82	[0·99-3·37]	0·055	1·08	[0·36-3·25]	0·89
Non sedentary	D1	1·07	[0·95-1·21]	0·25	1·13	[0·98-1·30]	0·102	0·94	[0·75-1·18]	0·58
	D2	1·14	[0·94-1·38]	0·17	1·03	[0·81-1·30]	0·822	1·24	[0·90-1·72]	0·20
OR = Odds ratio; CI = Confidence interval; IMD[1-3] = high deprivation, IMD[8-10] = low deprivation; reference category for IMD is the intermediate category IMD [4-7]; D1=After first dose; D2=After second dose<br>All the univariate models are adjusted by age, BMI, and sex, except Not obese that is adjusted by only age and sex										

Supplementary Table 7. Sensitivity analysis of univariate analyses for IMD categories, obesity status, and healthy lifestyle factors, using Inverse Probability Weighting (IPW) for probability of vaccination.

		All age groups			Younger adults (18-59 years)			Older adults (60+ years)		
		OR	95% CI	p-value	OR	95% CI	p-value	OR	95% CI	p-value
IMD[1-3]	D1	1·19	[1·08-1·30]	<0·0001	1·12	[1·00-1·25]	0·04	1·39	[1·15-1·68]	0·0010
	D2	1·00	[0·85-1·18]	0·98	0·86	[0·70-1·06]	0·16	1·28	[0·96-1·69]	0·091
IMD[8-10]	D1	0·91	[0·85-0·97]	0·0050	0·93	[0·86-1·01]	0·10	0·84	[0·75-0·95]	0·0050
	D2	0·94	[0·83-1·07]	0·37	0·95	[0·80-1·13]	0·55	0·93	[0·76-1·13]	0·46
Healthy lifestyle score	D1	0·97	[0·92-1·02]	0·26	0·99	[0·93-1·06]	0·85	0·92	[0·83-1·02]	0·12
	D2	1·02	[0·93-1·11]	0·71	0·97	[0·87-1·08]	0·58	1·07	[0·92-1·25]	0·35
Healthier diet	D1	0·95	[0·86-1·04]	0·25	0·93	[0·83-1·04]	0·20	0·92	[0·77-1·10]	0·35
	D2	0·95	[0·81-1·10]	0·46	0·84	[0·69-1·02]	0·074	1·09	[0·85-1·40]	0·50
Non obese	D1	0·86	[0·77-0·96]	0·0060	0·94	[0·82-1·06]	0·31	0·80	[0·64-1·00]	0·046
	D2	0·94	[0·79-1·11]	0·48	0·94	[0·76-1·16]	0·57	0·95	[0·70-1·29]	0·76
Non smoker	D1	1·13	[0·88-1·44]	0·34	1·09	[0·83-1·42]	0·53	1·24	[0·62-2·49]	0·54
	D2	1·55	[0·99-2·43]	0·055	1·91	[1·14-3·22]	0·014	0·71	[0·23-2·22]	0·56
Non sedentary	D1	1·08	[0·97-1·21]	0·18	1·14	[0·99-1·30]	0·064	0·95	[0·76-1·19]	0·68
	D2	1·16	[0·97-1·40]	0·099	1·04	[0·83-1·30]	0·73	1·32	[0·95-1·82]	0·10

OR = Odds ratio; CI = Confidence interval; IMD[1-3] = high deprivation, IMD[8-10] = low deprivation; reference category for IMD is the intermediate category IMD [4-7]; D1=After first dose; D2=After second dose.
All the univariate models are adjusted by age, BMI, and sex, except Not obese that is adjusted by only age and sex.

Supplementary Table 8. Multivariate analysis of frailty, IMD category, obesity status and healthy lifestyle factors, adjusted for age, BMI, and sex.

		All age groups			Younger adults (18-59 years)			Older adults (60+ years)		
		OR	95% CI	p-value	OR	95% CI	p-value	OR	95% CI	p-value
Frailty	D1	0·77	[0·57-1·06]	0·11	0·75	[0·48-1·16]	0·20	1·06	[0·65-1·73]	0·82
	D2	0·84	[0·52-1·34]	0·46	0·90	[0·44-1·83]	0·78	0·97	[0·50-1·87]	0·92
IMD[1-3]	D1	1·04	[0·91-1·20]	0·50	1·02	[0·87-1·19]	0·80	1·08	[0·81-1·44]	0·61
	D2	0·94	[0·75-1·16]	0·56	0·79	[0·61-1·04]	0·091	1·29	[0·87-1·93]	0·21
IMD[8-10]	D1	0·89	[0·81-1·00]	0·041	0·91	[0·80-1·03]	0·15	0·84	[0·69-1·02]	0·084
	D2	0·97	[0·783-1·14]	0·71	0·93	[0·76-1·14]	0·49	1·01	[0·78-1·32]	0·93
Healthier diet	D1	0·93	[0·84-1·03]	0·15	0·91	[0·81-1·03]	0·14	0·90	[0·75-1·08]	0·26
	D2	0·90	[0·78-1·05]	0·20	0·83	[0·68-1·01]	0·063	0·98	[0·77-1·26]	0·90
Non obese	D1	0·84	[0·75-0·95]	0·0040	1·16	[1·00-1·34]	0·049	0·87	[0·69-1·10]	0·24
	D2	0·87	[0·73-1·04]	0·13	1·06	[0·83-1·35]	0·085	0·93	[0·68-1·28]	0·66
Non smoker	D1	1·18	[0·88-1·57]	0·27	1·15	[0·84-1·58]	0·39	1·21	[0·57-2·58]	0·62
	D2	1·60	[0·95-2·69]	0·081	1·86	[1·00-3·46]	0·049	1·02	[0·34-3·05]	0·97
Non sedentary	D1	1·10	[0·98-1·25]	0·099	1·16	[1·00-1·34]	0·046	0·99	[0·79-1·26]	0·96
	D2	1·16	[0·96-1·40]	0·13	1·06	[0·83-1·34]	0·64	1·27	[0·91-1·78]	0·16

OR = Odds ratio; CI = Confidence interval; IMD[1-3] = high deprivation, IMD[8-10] = low deprivation; reference category for IMD is the intermediate category IMD [4-7]; D1=After first dose; D2=After second dose
All the univariate models are adjusted by age, BMI, and sex, except Not obese that is adjusted by only age and sex

Supplementary Table 9. Sensitivity analysis of the multivariate model for frailty, IMD categories, obesity status, and healthy lifestyle factors, using Inverse Probability Weighting (IPW) for probability of vaccination. The analysis is adjusted by age and sex.

		All age groups			Younger adults (18-59 years)			Older adults (60+ years)		
		OR	95% CI	p-value	OR	95% CI	p-value	OR	95% CI	p-value
Frailty	D1	0·77	[0·57-1·06]	0·11	1·15	[0·84-1·58]	0·39	1·21	[0·57-2·58]	0·62
	D2	0·84	[0·52-1·34]	0·46	1·86	[1·00-3·46]	0·049	1·02	[0·34-3·05]	0·97
IMD[1-3]	D1	1·05	[0·91-1·20]	0·50	1·16	[1·00-1·34]	0·046	0·99	[0·79-1·26]	0·96
	D2	0·94	[0·75-1·16]	0·56	1·06	[0·83-1·35]	0·64	1·27	[0·91-1·78]	0·16
IMD[8-10]	D1	0·90	[0·81-1·00]	0·04	0·91	[0·81-1·03]	0·14	0·90	[0·75-1·08]	0·26
	D2	0·97	[0·83-1·14]	0·71	0·83	[0·68-1·01]	0·063	0·98	[0·77-1·26]	0·90
Healthier diet	D1	0·93	[0·84-1·03]	0·15	0·91	[0·80-1·03]	0·15	0·84	[0·69-1·02]	0·084
	D2	0·90	[0·78-1·05]	0·19	0·93	[0·76-1·14]	0·49	1·01	[0·78-1·32]	0·93
Non obese	D1	0·84	[0·75-0·95]	<0·0001	1·02	[0·87-1·19]	0·80	1·08	[0·81-1·44]	0·61
	D2	0·87	[0·73-1·04]	0·13	0·79	[0·61-1·04]	0·091	1·29	[0·87-1·93]	0·21
Non smoker	D1	1·18	[0·88-1·57]	0·27	0·87	[0·76-1·00]	0·049	0·87	[0·69-1·10]	0·24
	D2	1·60	[0·95-2·69]	0·08	0·82	[0·66-1·03]	0·091	0·93	[0·68-1·28]	0·66
Non sedentary	D1	1·11	[0·98-1·25]	0·10	0·75	[0·48-1·16]	0·20	1·06	[0·65-1·73]	0·82
	D2	1·16	[0·96-1·40]	0·13	0·90	[0·44-1·83]	0·78	0·97	[0·50-1·87]	0·92

OR = Odds ratio; CI = Confidence interval; IMD[1-3] = high deprivation, IMD[8-10] = low deprivation; reference category for IMD is the intermediate category IMD [4-7]; D1=After first dose; D2=After second dose
All the univariate models are adjusted by age, BMI, and sex, except Not obese that is adjusted by only age and sex

Supplementary Table 10. Number and proportion of type of tests performed by individuals testing positive for in both cases (first and second dose vaccinated individuals) and controls (non-vaccinated individuals).

	**Cases-3 and Cases-4 n=3825 (D1) n=906 (D2)**			**Controls-3 and Controls-4 n=3825 (D1) n=906 (D2)**		
	RT-PCR confirmed tests n(%)	LFAT confirmed tests n(%)	Not sure n(%)	RT-PCR confirmed tests n(%)	LFAT confirmed tests n(%)	Not sure n(%)
D1	2666(70%)	927(24%)	231(6%)	1883(49%)	362(9%)	1580(41%)
D2	592(65%)	270(30%)	44(5%)	605(67%)	136(15%)	165(18%)

n = number of individuals; D1=After first dose; D2=After second dose Cases-3=cases infected after the first dose but before the second dose of the vaccine; Cases-4=cases infected after the second dose of the vaccine; Controls-3 and Controls-4=unvaccinated users tested positive after first and second dose matched with Cases-3 and Cases-4, respectively

Supplementary Table 11. Univariate analysis assessing the probability of asymptomatic infection, severe disease (>5 reported symptoms during acute infection), hospitalisation and duration of symptoms ≥28 days in app participants following first and second vaccination, adjusted by age, BMI, and sex.

		All age groups			Younger adults (18-59 years)			Older adults (60+ years)		
		OR	95% CI	p-value	OR	95% CI	p-value	OR	95% CI	p-value
Hospitalisation	D1	0·31	[0·25-0·38]	<0·0001	0·43	[0·32-0·58]	<0·0001	0·24	[0·18-0·31]	<0·0001
	D2	0·27	[0·16-0·45]	<0·0001	0·57	[0·26-1·23]	0·15	0·15	[0·07-0·32]	<0·0001
>5 reported symptoms	D1	0·74	[0·65-0·84]	<0·0001	0·77	[0·65-0·91]	0·0030	0·68	[0·55-0·84]	<0·0001
	D2	0·69	[0·51-0·94]	0·0180	0·70	[0·46-1·06]	0·093	0·66	[0·42-1·03]	0·067
symptoms lasting ≥28 days	D1	1·03	[0·85-1·24]	0·78	1·22	[0·94-1·60]	0·14	0·87	[0·67-1·13]	0·29
	D2	0·51	[0·32-0·82]	0·0060	0·37	[0·16-0·88]	0·025	0·56	[0·31-0·98]	0·044
Asymptomatic infection	D1	1·63	[1·43-1·86]	<0·0001	1·36	[1·14-1·62]	<0·0001	2·06	[1·68-2·52]	<0·0001
	D2	1·94	[1·49-2·54]	<0·0001	1·49	[1·00-2·24]	0·050	2·38	[1·66-3·41]	<0·0001

OR = Odds ratio; CI = Confidence interval; BMI = Body mass index; D1=After first dose; D2=After second dose

Supplementary Table 12. Numbers and proportions of individual symptoms in app participants testing positive following first and second vaccination, and in unvaccinated controls.

		Cases-3 and Cases-4			Controls-3 and Controls-4		
		**All n=3825 (D1) n=906(D2)**	**[18-59 years] n=2320 (D1) n=455 (D2)**	**[60+ years] n=1505 (D1) n=451(D2)**	**All n=3285 (D1) n=906(D2)**	**[18-59 years] n=2363 (D1) n=474(D2)**	**[60+ years] n= 1462 (D1) n=432(D2)**
Fever n(%)	D1	2525(68·6)	1628(72·9)	897(61·9)	2825(82·0)	1760(83·0)	1065(80·4)
	D2	549(61·9)	308(69·4)	241(54·4)	689(82·5)	360(84·7)	329(80·2)
Persistent cough n(%)	D1	1200(32·6)	780(34·9)	420(29·0)	1328(38·5)	806(38·0)	522(39·4)
	D2	248(28·0)	138(31·1)	110(24·8)	335(40·1)	172(40·5)	163(39·8)
Loss of smell n(%)	D1	841(22·8)	581(26·0)	260(17·9)	1591(46·2)	962(45·4)	629(47·5)
	D2	184(20·7)	115(25·9)	69(15·6)	413(49·5)	203(47·8)	210(51·2)
Fatigue n(%)	D1	1798(48·8)	1178(52·7)	620(42·8)	2246(65·2)	1378(65·0)	868(65·6)
	D2	388(43·7)	219(49·3)	169(38·1)	548(65·6)	270(63·5)	278(67·8)
Headache n(%)	D1	1795(48·7)	1228(55·0)	567(39·1)	2158(62·6)	1414(66·7)	744(56·2)
	D2	369(41·6)	237(53·4)	132(29·8)	507(60·7)	270(63·5)	237(57·8)
Runny nose n(%)	D1	1779(48·3)	1198(53·6)	581(40·1)	2059(59·8)	1307(61·6)	752(56·8)
	D2	381(43·0)	225(50·7)	156(35·2)	513(61·4)	261(61·4)	252(61·5)
Sneezing n(%)	D1	1560(42·4)	1053(47·1)	507(35·0)	1368(39·7)	873(41·2)	495(37·4)
	D2	346(39·0)	204(45·9)	142(32·1)	354(42·4)	179(42·1)	175(42·7)
Sore throat n(%)	D1	1229(33·4)	859(38·5)	370(25·5)	1583(46·0)	1055(49·7)	528(39·9)
	D2	260(29·3)	161(36·3)	99(22·3)	400(47·9)	211(49·6)	189(46·1)
	D1	883(24·0)	593(26·5)	290(20·0)	1071(31·1)	681(32·1)	390(29·5)

Dizziness or lightheadedness n(%)	D2	177(20·0)	115(25·9)	62(14·0)	240(28·7)	129(30·4)	111(27·1)
Chills or shivers n(%)	D1	815(22·1)	519(23·2)	296(20·4)	1164(33·8)	717(33·8)	447(33·8)
	D2	152(17·1)	76(17·1)	76(17·2)	251(30·1)	126(29·6)	125(30·5)
Hoarse voice n(%)	D1	913(24·8)	597(26·7)	316(21·8)	1073(31·1)	679(32·0)	394(29·8)
	D2	182(20·5)	101(22·7)	81(18·3)	283(33·9)	143(33·6)	140(34·1)
Skipped meals n(%)	D1	623(16·9)	402(18·0)	221(15·3)	914(26·5)	518(24·4)	396(29·9)
	D2	138(15·6)	80(18·0)	58(13·1)	210(25·1)	107(25·2)	103(25·1)
Brain fog n(%)	D1	755(20·5)	536(24·0)	219(15·1)	769(22·3)	500(23·6)	269(20·3)
	D2	150(16·9)	89(20·0)	61(13·8)	173(20·7)	90(21·2)	83(20·2)
Unusual muscle pains n(%)	D1	639(17·3)	442(19·8)	197(13·6)	762(22·1)	526(24·8)	236(17·8)
	D2	126(14·2)	82(18·5)	44(9·9)	156(18·7)	99(23·3)	57(13·9)
Eye soreness n(%)	D1	677(18·4)	463(20·7)	214(14·8)	857(24·9)	567(26·7)	290(21·9)
	D2	154(17·4)	93(20·9)	61(13·8)	216(25·9)	125(29·4)	91(22·2)
Diarrhoea n(%)	D1	485(13·2)	317(14·2)	168(11·6)	766(22·2)	453(21·4)	313(23·6)
	D2	92(10·4)	58(13·1)	34(7·7)	175(21·1)	88(20·7)	87(21·2)
Shortness of breath n(%)	D1	605(16·4)	410(18·4)	195(13·5)	566(16·4)	340(16·0)	226(17·1)
	D2	102(11·5)	65(14·6)	37(8·4)	118(14·1)	62(14·6)	56(13·7)
Low mood n(%)	D1	508(13·8)	346(15·5)	162(11·2)	677(19·7)	387(18·2)	290(21·9)
	D2	98(11·0)	66(14·9)	32(7·2)	173(20·7)	83(19·5)	90(22·0)
Chest pain n(%)	D1	601(16·3)	427(19·1)	174(12·0)	610(17·7)	400(18·9)	210(15·9)
	D2	105(11·8)	67(15·1)	38(8·6)	124(14·9)	64(15·1)	60(14·6)
Nausea n(%)	D1	433(11·8)	293(13·1)	140(9·7)	641(18·6)	391(18·4)	250(18·9)
	D2	97(10·9)	67(15·1)	30(6·8)	124(14·9)	65(15·3)	59(14·4)
Tinnitus n(%)	D1	452(12·3)	291(13·0)	161(11·1)	501(14·5)	306(14·4)	195(14·7)
	D2	89(10·0)	52(11·7)	37(8·4)	129(15·4)	58(13·6)	71(17·3)
Abdominal pain n(%)	D1	396(10·8)	260(11·6)	136(9·4)	493(14·3)	310(14·6)	183(13·8)
	D2	80(9·0)	54(12·2)	26(5·9)	116(13·9)	62(14·6)	54(13·2)
Earache n(%)	D1	423(11·5)	321(14·4)	102(7·0)	416(12·1)	285(13·4)	131(9·9)
	D2	87(9·8)	61(13·7)	26(5·9)	98(11·7)	58(13·6)	40(9·8)
Swollen glands n(%)	D1	449(12·2)	329(14·7)	120(8·3)	413(12·0)	287(13·5)	126(9·5)
	D2	110(12·4)	79(17·8)	31(7·0)	97(11·6)	58(13·6)	39(9·5)
Sensation of skin n(%)burning	D1	260(7·1)	184(8·2)	76(5·2)	472(13·7)	315(14·9)	157(11·9)
	D2	52(5·9)	32(7·2)	20(4·5)	99(11·9)	57(13·4)	42(10·2)
Delirium or reported confusion n(%)	D1	338(9·2)	213(9·5)	125(8·6)	396(11·5)	237(11·2)	159(12·0)
	D2	59(6·7)	37(8·3)	22(5·0)	84(10·1)	41(9·6)	43(10·5)
Irregular heartbeat n(%)	D1	264(7·2)	187(8·4)	77(5·3)	344(10·0)	219(10·3)	125(9·4)
	D2	55(6·2)	33(7·4)	22(5·0)	79(9·5)	38(8·9)	41(10·0)
typical hayfever n(%)	D1	273(7·4)	213(9·5)	60(4·1)	364(10·6)	229(10·8)	135(10·2)
	D2	96(10·8)	74(16·7)	22(5·0)	116(13·9)	60(14·1)	56(13·7)
rash n(%)	D1	112(3·0)	62(2·8)	50(3·5)	167(4·8)	98(4·6)	69(5·2)
	D2	25(2·8)	14(3·2)	11(2·5)	37(4·4)	17(4·0)	20(4·9)
	D1	112(3·0)	62(2·8)	50(3·5)	167(4·8)	98(4·6)	69(5·2)

red welts on face or lips n(%)	D2	14(1·6)	10(2·3)	4(0·9)	32(3·8)	15(3·5)	17(4·1)
blisters on feet n(%)	D1	76(2·1)	52(2·3)	24(1·7)	142(4·1)	80(3·8)	62(4·7)
	D2	7(0·8)	3(0·7)	4(0·9)	6(0·7)	2(0·5)	4(1·0)
hair loss n(%)	D1	30(0·8)	12(0·5)	18(1·2)	28(0·8)	12(0·6)	16(1·2)
	D2	5(0·6)	3(0·7)	2(0·5)	10(1·2)	5(1·2)	5(1·2)

n=number of individuals; D1=After first dose; D2=After second dose; Cases-3=cases infected after the first dose but before the second dose of the vaccine; Cases-4=cases infected after the second dose of the vaccine; Controls-3 and Controls-4=unvaccinated users tested positive after first and second dose matched with Cases-3 and Cases-4, respectively
Symptoms are ordered by frequency, core symptoms highlighted in gray ((https://www·nhs·uk/conditions/coronavirus-covid-19/symptoms/main-symptoms/)

Supplementary Table 13. Univariate analysis assessing the probability of experiencing each symptom in app participants following first and second vaccination, adjusted by age, BMI, sex.

		All age groups			Younger adults (18-59 years)			Older adults (60+ years)		
		OR	95% CI	p-value	OR	95% CI	p-value	OR	95% CI	p-value
Fever	D1	0·35	[0·31-0·38]	<0·0001	0·42	[0·37-0·48]	<0·0001	0·24	[0·20-0·28]	<0·0001
	D2	0·27	[0·22-0·33]	<0·0001	0·38	[0·29-0·51]	<0·0001	0·17	[0·13-0·24]	<0·0001
Persistent cough	D1	0·77	[0·70-0·85]	<0·0001	0·87	[0·77-0·99]	0·032	0·63	[0·53-0·73]	<0·0001
	D2	0·57	[0·47-0·70]	<0·0001	0·65	[0·49-0·86]	<0·0001	0·5	[0·38-0·68]	<0·0001
Loss of smell	D1	0·48	[0·43-0·54]	<0·0001	0·55	[0·47-0·63]	<0·0001	0·4	[0·33-0·47]	<0·0001
	D2	0·34	[0·27-0·43]	<0·0001	0·4	[0·29-0·56]	<0·0001	0·3	[0·22-0·40]	<0·0001
Fatigue	D1	0·51	[0·46-0·56]	<0·0001	0·6	[0·53-0·68]	<0·0001	0·39	[0·34-0·46]	<0·0001
	D2	0·41	[0·33-0·49]	<0·0001	0·55	[0·42-0·72]	<0·0001	0·29	[0·22-0·39]	<0·0001
Headache	D1	0·56	[0·51-0·62]	<0·0001	0·6	[0·53-0·68]	<0·0001	0·49	[0·42-0·57]	<0·0001
	D2	0·45	[0·37-0·55]	<0·0001	0·63	[0·48-0·83]	<0·0001	0·3	[0·23-0·41]	<0·0001
Runny nose	D1	0·63	[0·57-0·69]	<0·0001	0·72	[0·63-0·81]	<0·0001	0·5	[0·43-0·59]	<0·0001
	D2	0·47	[0·39-0·57]	<0·0001	0·63	[0·48-0·83]	<0·0001	0·34	[0·26-0·45]	<0·0001
Sneezing	D1	1·12	[1·02-1·23]	0·023	1·27	[1·13-1·43]	<0·0001	0·91	[0·78-1·06]	0·23
	D2	0·87	[0·72-1·06]	0·17	1·15	[0·88-1·51]	0·31	0·65	[0·49-0·86]	<0·0001
Sore throat	D1	0·59	[0·53-0·65]	<0·0001	0·63	[0·56-0·71]	<0·0001	0·51	[0·44-0·61]	<0·0001
	D2	0·45	[0·36-0·54]	<0·0001	0·56	[0·43-0·74]	<0·0001	0·34	[0·25-0·46]	<0·0001
Dizziness /lightheadedness	D1	0·7	[0·63-0·77]	<0·0001	0·76	[0·67-0·87]	<0·0001	0·6	[0·50-0·71]	<0·0001
	D2	0·61	[0·49-0·76]	<0·0001	0·78	[0·58-1·05]	0·10	0·44	[0·31-0·63]	<0·0001
Chills or shivers	D1	0·56	[0·50-0·62]	<0·0001	0·59	[0·51-0·67]	<0·0001	0·5	[0·42-0·60]	<0·0001
	D2	0·48	[0·38-0·60]	<0·0001	0·49	[0·35-0·68]	<0·0001	0·47	[0·34-0·66]	<0·0001
Hoarse voice	D1	0·73	[0·66-0·81]	<0·0001	0·77	[0·67-0·88]	<0·0001	0·66	[0·56-0·78]	<0·0001
	D2	0·5	[0·40-0·62]	<0·0001	0·56	[0·41-0·76]	<0·0001	0·43	[0·32-0·60]	<0·0001
Skipped meals	D1	0·56	[0·50-0·63]	<0·0001	0·67	[0·58-0·78]	<0·0001	0·41	[0·34-0·50]	<0·0001
	D2	0·54	[0·43-0·69]	<0·0001	0·62	[0·45-0·87]	0·012	0·44	[0·31-0·63]	<0·0001
Brain fog	D1	0·90	[0·80-1·01]	0·074	1·02	[0·89-1·18]	0·76	0·7	[0·57-0·85]	<0·0001
	D2	0·77	[0·61-0·99]	0·041	0·9	[0·65-1·26]	0·54	0·63	[0·44-0·91]	0·012

Unusual muscle pains	D1	0·74	[0·66-0·83]	<0·0001	0·74	[0·64-0·86]	<0·0001	0·73	[0·59-0·89]	<0·0001
	D2	0·72	[0·55-0·93]	0·010	0·73	[0·52-1·01]	0·061	0·69	[0·46-1·06]	0·091
Eye soreness	D1	0·68	[0·61-0·76]	<0·0001	0·71	[0·62-0·82]	<0·0001	0·62	[0·51-0·75]	<0·0001
	D2	0·6	[0·47-0·75]	<0·0001	0·62	[0·45-0·84]	<0·0001	0·57	[0·40-0·81]	<0·0001
Diarrhoea	D1	0·52	[0·46-0·59]	<0·0001	0·6	[0·51-0·71]	<0·0001	0·42	[0·34-0·51]	<0·0001
	D2	0·42	[0·32-0·56]	<0·0001	0·55	[0·38-0·79]	<0·0001	0·31	[0·20-0·47]	<0·0001
Shortness of breath	D1	0·42	[0·32-0·56]	<0·0001	0·55	[0·38-0·79]	<0·0001	0·31	[0·20-0·47]	<0·0001
	D2	0·79	[0·59-1·04]	0·10	0·97	[0·66-1·41]	0·87	0·56	[0·36-0·87]	0·011
Low mood	D1	0·65	[0·57-0·74]	<0·0001	0·82	[0·70-0·96]	0·014	0·44	[0·36-0·55]	<0·0001
	D2	0·47	[0·36-0·61]	<0·0001	0·69	[0·49-0·99]	0·042	0·28	[0·18-0·43]	<0·0001
Chest pain	D1	0·91	[0·80-1·03]	0·13	1·02	[0·87-1·18]	0·84	0·73	[0·58-0·90]	<0·0001
	D2	0·77	[0·58-1·02]	0·071	1	[0·69-1·46]	0·99	0·56	[0·36-0·86]	0·011
Nausea	D1	0·58	[0·50-0·66]	<0·0001	0·66	[0·56-0·78]	<0·0001	0·45	[0·36-0·57]	<0·0001
	D2	0·69	[0·52-0·92]	0·011	0·95	[0·66-1·38]	0·79	0·43	[0·27-0·69]	<0·0001
Tinnitus	D1	0·82	[0·71-0·94]	<0·0001	0·88	[0·74-1·04]	0·14	0·73	[0·58-0·91]	0·018
	D2	0·6	[0·45-0·81]	<0·0001	0·82	[0·55-1·22]	0·33	0·44	[0·29-0·68]	<0·0001
Abdominal pain	D1	0·72	[0·62-0·83]	<0·0001	0·76	[0·64-0·91]	<0·0001	0·65	[0·51-0·82]	<0·0001
	D2	0·6	[0·44-0·82]	<0·0001	0·77	[0·52-1·15]	0·20	0·42	[0·25-0·68]	<0·0001
Earache	D1	0·94	[0·82-1·09]	0·44	1·07	[0·90-1·27]	0·44	0·69	[0·52-0·90]	0·014
	D2	0·81	[0·59-1·10]	0·17	0·95	[0·64-1·41]	0·81	0·59	[0·35-0·99]	0·042
Swollen glands	D1	1·02	[0·89-1·18]	0·74	1·1	[0·93-1·31]	0·27	0·86	[0·66-1·12]	0·26
	D2	1·06	[0·79-1·43]	0·68	1·32	[0·91-1·92]	0·15	0·73	[0·45-1·21]	0·22
Sensation of skin burning	D1	0·48	[0·41-0·56]	<0·0001	0·51	[0·42-0·62]	<0·0001	0·41	[0·31-0·55]	<0·0001
	D2	0·47	[0·33-0·66]	<0·0001	0·5	[0·32-0·79]	<0·0001	0·42	[0·24-0·73]	<0·0001
Delirium or reported confusion	D1	0·77	[0·66-0·90]	<0·0001	0·84	[0·69-1·02]	0·080	0·66	[0·52-0·86]	<0·0001
	D2	0·61	[0·43-0·87]	0·010	0·78	[0·49-1·26]	0·32	0·41	[0·24-0·70]	<0·0001
Irregular heartbeat	D1	0·69	[0·59-0·82]	<0·0001	0·79	[0·64-0·97]	0·020	0·54	[0·40-0·72]	<0·0001
	D2	0·62	[0·43-0·89]	0·010	0·79	[0·49-1·29]	0·35	0·48	[0·28-0·82]	0·017

OR = Odds ratio; CI = Confidence interval; D1=After first dose; D2=After second dose

Supplementary Table 14. Univariate analysis assessing the probability of asymptomatic infection, severe disease (>5 reported symptoms during acute infection), hospitalisation, and duration of symptoms ≥ 28 days in app participants following first and second vaccination, adjusted by age, BMI, sex, frailty, and presence of at least one comorbidity.

		All age groups			Younger adults (18-59 years)			Older adults (60+ years)		
		OR	95% CI	p-value	OR	95% CI	p-value	OR	95% CI	p-value
Hospitalisation	D1	0·29	[0·24-0·36]	<0·0001	0·42	[0·31-0·57]	<0·0001	0·22	[0·17-0·29]	<0·0001
	D2	0·26	[0·15-0·43]	<0·0001	0·52	[0·24-1·13]	0·10	0·15	[0·07-0·31]	<0·0001
>5 reported symptoms	D1	0·72	[0·64-0·83]	<0·0001	0·76	[0·64-0·90]	0·0010	0·66	[0·53-0·82]	<0·0001
	D2	0·65	[0·48-0·89]	0·0070	0·59	[0·38-0·90]	0·016	0·64	[0·41-1·00]	0·048
symptoms lasting ≥28 days	D1	1·04	[0·86-1·25]	0·691	1·2	[0·92-1·57]	0·18	0·88	[0·68-1·15]	0·353
	D2	0·51	[0·32-0·82]	0·005	0·21	[0·08-0·59]	0·003	0·58	[0·33-1·04]	0·067
Asymptomatic infection	D1	1·62	[1·42-1·86]	<0·0001	1·35	[1·13-1·61]	<0·0001	2·06	[1·69-2·53]	<0·0001
	D2	1·94	[1·48-2·54]	<0·0001	1·49	[0·99-2·25]	0·06	2·37	[1·65-3·41]	<0·0001

OR = Odds ratio; CI = Confidence interval; BMI = Body mass index; D1=After first dose; D2=After second dose

Supplementary Table 15. Univariate analysis assessing the probability of experiencing each symptom in app participants following first and second vaccine dose, adjusted by age, BMI, sex, frailty, and presence of at least one comorbidity.

		All age groups			Younger adults (18-59 years)			Older adults (60+ years)		
		OR	95% CI	p-value	OR	95% CI	p-value	OR	95% CI	p-value
Fever	D1	0·35	[0·31-0·38]	<0·0001	0·42	[0·37-0·48]	<0·0001	0·24	[0·20-0·28]	<0·0001
	D2	0·27	[0·21-0·33]	<0·0001	0·36	[0·27-0·49]	<0·0001	0·17	[0·12-0·24]	<0·0001
Persistent cough	D1	0·77	[0·69-0·85]	<0·0001	0·87	[0·77-0·98]	0·030	0·63	[0·53-0·73]	<0·0001
	D2	0·57	[0·46-0·70]	<0·0001	0·62	[0·47-0·83]	0·0010	0·51	[0·38-0·69]	<0·0001
Loss of smell	D1	0·48	[0·43-0·54]	<0·0001	0·55	[0·47-0·64]	<0·0001	0·39	[0·33-0·47]	<0·0001
	D2	0·34	[0·27-0·42]	<0·0001	0·39	[0·28-0·55]	<0·0001	0·29	[0·21-0·40]	<0·0001
Fatigue	D1	0·51	[0·46-0·56]	<0·0001	0·59	[0·53-0·67]	<0·0001	0·39	[0·34-0·46]	<0·0001
	D2	0·45	[0·37-0·55]	<0·0001	0·62	[0·47-0·82]	0·0010	0·3	[0·23-0·41]	<0·0001
Headache	D1	0·57	[0·51-0·62]	<0·0001	0·6	[0·53-0·68]	<0·0001	0·49	[0·42-0·57]	<0·0001
	D2	0·45	[0·37-0·55]	<0·0001	0·62	[0·47-0·82]	0·0010	0·3	[0·23-0·41]	<0·0001
Runny nose	D1	0·63	[0·58-0·70]	<0·0001	0·72	[0·64-0·81]	<0·0001	0·5	[0·43-0·59]	<0·0001
	D2	0·47	[0·39-0·57]	<0·0001	0·62	[0·47-0·82]	0·0010	0·34	[0·25-0·45]	<0·0001
Sneezing	D1	1·14	[1·03-1·25]	0·010	1·28	[1·13-1·44]	<0·0001	0·92	[0·78-1·07]	0·28
	D2	0·88	[0·72-1·07]	0·19	1·16	[0·89-1·53]	0·28	0·64	[0·48-0·85]	0·0020
Sore throat	D1	0·59	[0·54-0·65]	<0·0001	0·63	[0·56-0·71]	<0·0001	0·51	[0·44-0·60]	<0·0001
	D2	0·44	[0·36-0·54]	<0·0001	0·55	[0·42-0·72]	<0·0001	0·34	[0·25-0·46]	<0·0001
Dizziness /lightheadedness	D1	0·7	[0·63-0·77]	<0·0001	0·75	[0·66-0·86]	<0·0001	0·6	[0·50-0·71]	<0·0001
	D2	0·6	[0·48-0·75]	<0·0001	0·75	[0·55-1·02]	0·063	0·44	[0·31-0·63]	<0·0001
Chills or shivers	D1	0·56	[0·50-0·62]	<0·0001	0·58	[0·51-0·67]	<0·0001	0·5	[0·42-0·60]	<0·0001
	D2	0·47	[0·37-0·59]	<0·0001	0·46	[0·33-0·64]	<0·0001	0·47	[0·34-0·65]	<0·0001
Hoarse voice	D1	0·73	[0·66-0·81]	<0·0001	0·77	[0·67-0·88]	<0·0001	0·66	[0·56-0·79]	<0·0001
	D2	0·5	[0·40-0·62]	<0·0001	0·53	[0·39-0·72]	<0·0001	0·44	[0·32-0·60]	<0·0001
Skipped meals	D1	0·55	[0·49-0·61]	<0·0001	0·67	[0·58-0·78]	<0·0001	0·4	[0·33-0·48]	<0·0001
	D2	0·52	[0·41-0·66]	<0·0001	0·61	[0·44-0·85]	0·0040	0·42	[0·29-0·61]	<0·0001
Brain fog	D1	0·89	[0·79-0·99]	0·040	1·01	[0·88-1·17]	0·86	0·68	[0·56-0·83]	<0·0001
	D2	0·75	[0·58-0·96]	0·020	0·87	[0·62-1·22]	0·42	0·63	[0·44-0·91]	0·013
Unusual muscle pains	D1	0·73	[0·65-0·82]	<0·0001	0·74	[0·64-0·85]	<0·0001	0·71	[0·58-0·88]	<0·0001
	D2	0·7	[0·54-0·91]	0·0080	0·68	[0·48-0·95]	0·024	0·69	[0·45-1·05]	0·080
Eye soreness	D1	0·68	[0·60-0·76]	<0·0001	0·71	[0·62-0·82]	<0·0001	0·62	[0·51-0·75]	<0·0001
	D2	0·58	[0·46-0·74]	<0·0001	0·6	[0·43-0·82]	0·0010	0·57	[0·40-0·82]	0·0020
Diarrhoea	D1	0·52	[0·46-0·59]	<0·0001	0·59	[0·51-0·70]	<0·0001	0·41	[0·33-0·51]	<0·0001
	D2	0·41	[0·31-0·54]	<0·0001	0·5	[0·35-0·74]	<0·0001	0·31	[0·20-0·47]	<0·0001
Shortness of breath	D1	0·96	[0·85-1·09]	0·56	1·15	[0·98-1·35]	0·090	0·71	[0·57-0·88]	<0·0001
	D2	0·72	[0·54-0·96]	0·027	0·85	[0·58-1·26]	0·43	0·54	[0·35-0·86]	0·0080
Low mood	D1	0·64	[0·57-0·73]	<0·0001	0·82	[0·70-0·96]	0·010	0·44	[0·36-0·54]	<0·0001
	D2	0·46	[0·35-0·60]	<0·0001	0·68	[0·47-0·97]	0·035	0·28	[0·18-0·43]	<0·0001

Chest pain	D1	0·9	[0·80-1·02]	0·10	1·01	[0·86-1·17]	0·93	0·72	[0·58-0·89]	<0·0001
	D2	0·74	[0·56-0·99]	0·041	0·94	[0·65-1·38]	0·76	0·56	[0·36-0·86]	0·0080
Nausea	D1	0·57	[0·50-0·65]	<0·0001	0·65	[0·55-0·76]	<0·0001	0·45	[0·36-0·56]	<0·0001
	D2	0·65	[0·48-0·86]	0·0030	0·87	[0·60-1·28]	0·49	0·42	[0·26-0·67]	<0·0001
Tinnitus	D1	0·83	[0·72-0·95]	0·010	0·87	[0·73-1·04]	0·13	0·74	[0·59-0·92]	0·010
	D2	0·59	[0·44-0·79]	<0·0001	0·77	[0·51-1·16]	0·21	0·45	[0·29-0·69]	<0·0001
Abdominal pain	D1	0·71	[0·62-0·82]	<0·0001	0·75	[0·63-0·89]	<0·0001	0·65	[0·51-0·82]	<0·0001
	D2	0·6	[0·44-0·81]	0·0010	0·72	[0·48-1·08]	0·12	0·43	[0·26-0·70]	0·0010
Earache	D1	0·94	[0·81-1·09]	0·41	1·06	[0·89-1·26]	0·51	0·69	[0·52-0·90]	0·010
	D2	0·79	[0·58-1·08]	0·14	0·92	[0·62-1·37]	0·69	0·59	[0·35-1·00]	0·050
Swollen glands	D1	1·03	[0·89-1·19]	0·72	1·1	[0·93-1·31]	0·27	0·86	[0·66-1·12]	0·26
	D2	1·05	[0·78-1·41]	0·75	1·28	[0·88-1·87]	0·20	0·75	[0·45-1·23]	0·25
Sensation of skin burning	D1	0·48	[0·41-0·56]	<0·0001	0·5	[0·41-0·61]	<0·0001	0·42	[0·31-0·56]	<0·0001
	D2	0·46	[0·32-0·65]	<0·0001	0·45	[0·29-0·72]	0·0010	0·43	[0·25-0·75]	0·0030
Delirium or reported confusion	D1	0·74	[0·63-0·86]	<0·0001	0·83	[0·68-1·01]	0·060	0·62	[0·48-0·81]	<0·0001
	D2	0·55	[0·38-0·79]	0·0010	0·72	[0·45-1·17]	0·19	0·36	[0·20-0·63]	<0·0001
Irregular heartbeat	D1	0·68	[0·57-0·80]	<0·0001	0·77	[0·63-0·95]	0·010	0·52	[0·39-0·70]	<0·0001
	D2	0·6	[0·42-0·86]	0·0060	0·72	[0·44-1·19]	0·20	0·47	[0·27-0·81]	0·0070
OR = Odds ratio; CI = Confidence interval										

Supplementary Figure 1. Inclusion process for cases and group controls

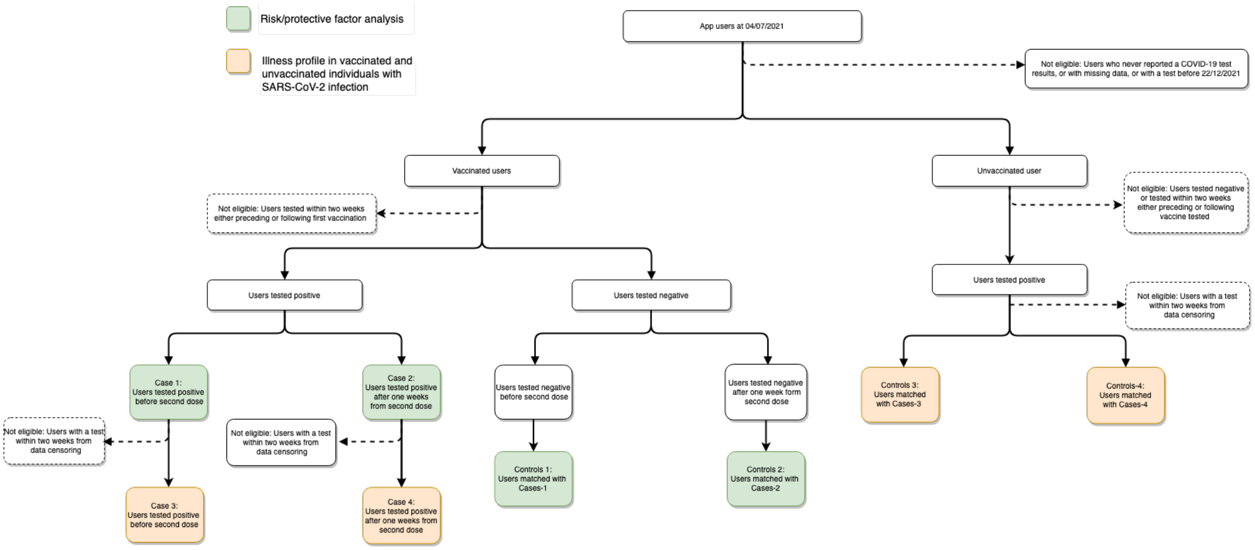

Supplementary Figure 2. Odds ratio of receiving the vaccine for all users who have recorded at least one COVID-19 test since 8th of December 2020. Univariate analysis for a) age, body mass index (BMI), presence of at least one comorbidity, and frailty; and b) Index of Multiple Deprivation category and healthy lifestyle factors, adjusted for age, BMI, and sex and stratified by age group.

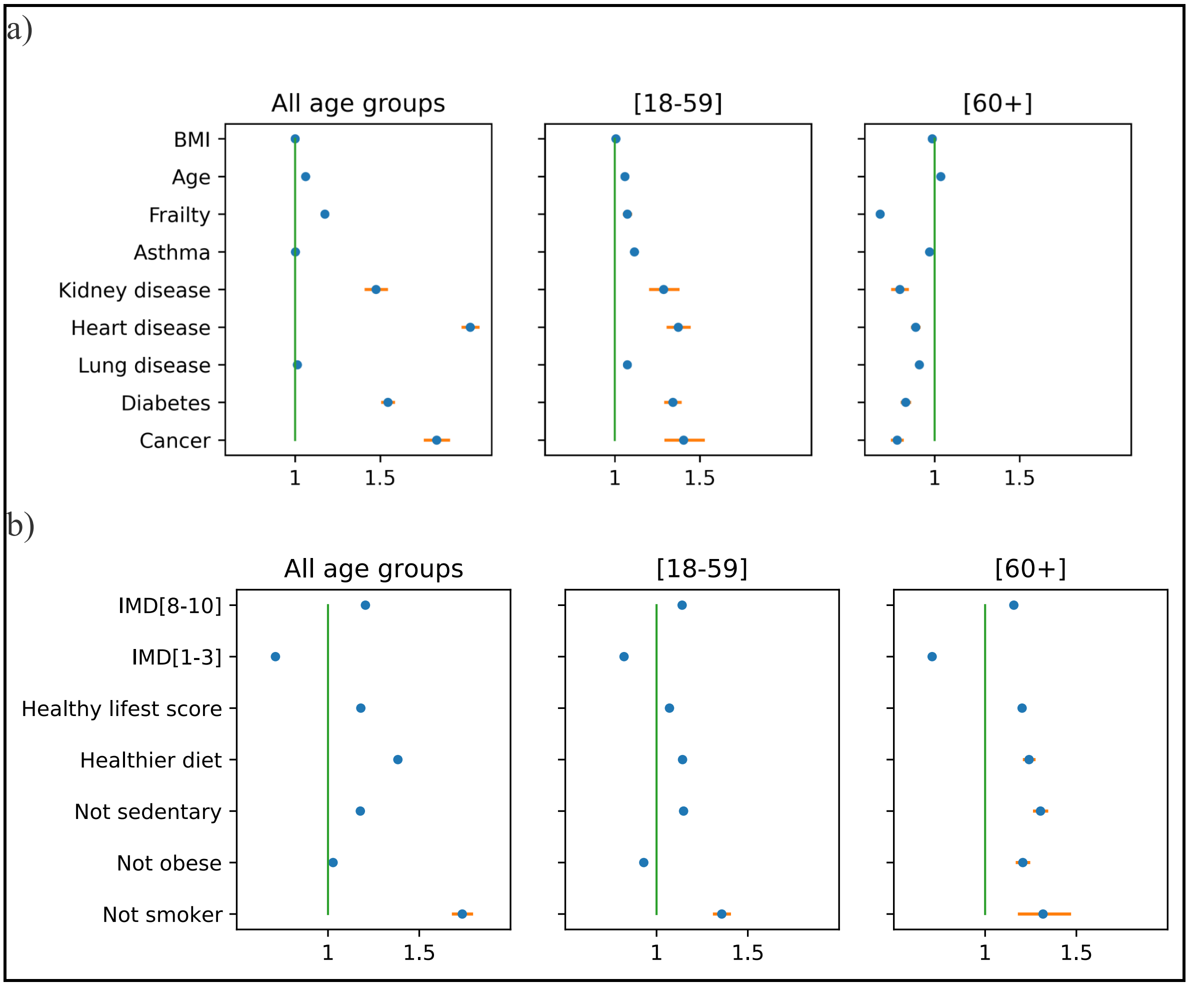

IMD = Index of Multiple Deprivation;IMD[1-3] = high deprivation, IMD[8-10]= low deprivation; reference category for IMD is the intermediate category IMD[4-7]

Supplementary Figure 3. Histogram illustrating number of reported positive tests against number of days from first (blue bar) and second (orange bar) vaccine dose and for COVID-19 infection. NB these data are not adjusted for incidence of infection which changed over the same time period in the UK.[46]

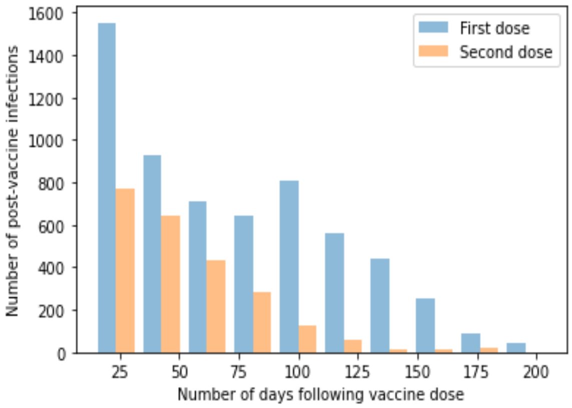

Supplementary Figure 4. Comparison between the original univariate model adjusted for age, BMI, sex (blue) and the univariate model adjusted by age, BMI, sex, and mask-wearing (orange) for a) Cases-1 and b) Cases-2.

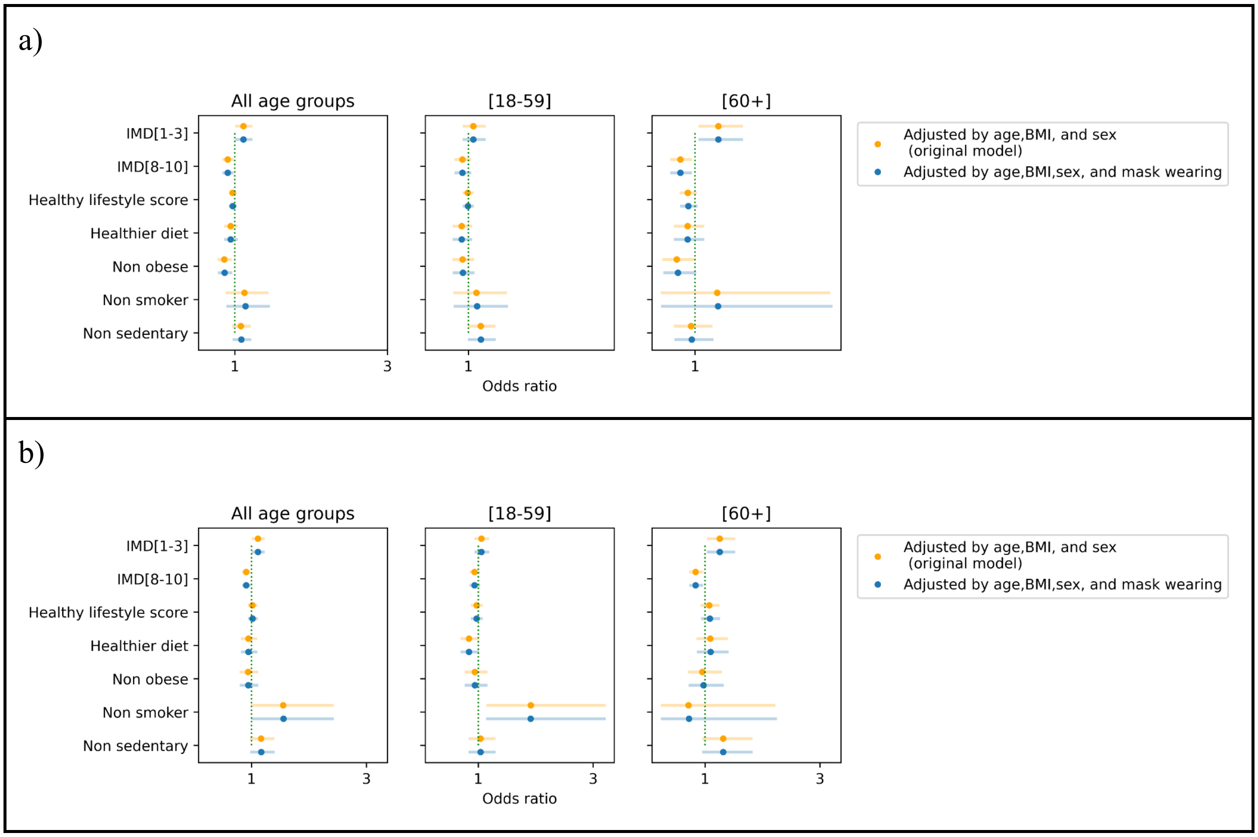

Supplementary Methods

Dietary Assessment

Diet was assessed using information obtained from an amended version of the Leeds Short Form Food Frequency Questionnaire that included 27 food items. Participants were asked how often on average they had consumed one portion of each food in a typical week during the month just prior (July 2020) to when they filled out the diet and lifestyle questionnaire. The responses had eight frequency categories ranging from "rarely or never" to "five or more times per day". A healthy diet pattern was ascertained using the Diet Quality Score (DQS), a validated score for adherence to UK dietary guidelines.[1,2] The DQS was computed from five broad categories including fruits, vegetables, total fat, oily fish, and non-milk extrinsic sugars. Each component was scored from 1 (unhealthiest) to 3 (healthiest) points, with intermediate values scored proportionally. All component scores were summed to obtain a total score ranging from 5 (lowest diet quality) to 15 (highest) points. We defined a healthier diet pattern as a DQS in the top quartile of the score distribution (score>=12 points). To generate the lifestyle scores, the participants received 1 point for each healthy lifestyle factor. The sum of these four scores together gave a healthy lifestyle score ranging from 0 to 4, with higher scores indicating a healthier lifestyle.[2]

1. Cleghorn CL, Harrison RA, Ransley JK, Wilkinson S, Thomas J, Cade JE. Can a dietary quality score derived from a short-form FFQ assess dietary quality in UK adult population surveys? *Public Health Nutr* 2016; **19**(16): 2915-23.
2. Merino J, Joshi AD, Nguyen L, Mazidi M, Graham M, Murray B, et al., Adherence to Healthy Diet and Risk and Severity of SARS-CoV-2 Infection: A Community Survey Study Within the COVID Symptom Study Application. American Society for Nutrition, June 7-10, 2021.
